# Extracellular Acidification Inhibits the ROS-Dependent Formation of Neutrophil Extracellular Traps

**DOI:** 10.3389/fimmu.2017.00184

**Published:** 2017-02-28

**Authors:** Martina Behnen, Sonja Möller, Antonia Brozek, Matthias Klinger, Tamás Laskay

**Affiliations:** ^1^Department for Infectious Diseases and Microbiology, University of Lübeck, Lübeck, Germany; ^2^Institute of Anatomy, University of Lübeck, Lübeck, Germany

**Keywords:** neutrophil extracellular traps, extracellular acidosis, pH, immobilized immune complexes, reactive oxygen species, metabolism, glycolysis

## Abstract

The inflammatory microenvironment is commonly characterized by extracellular acidosis (pH < 7.35). Sensitivity to pH, CO_2_ or bicarbonate concentrations allows neutrophils to react to changes in their environment and to detect inflamed areas in the tissue. One important antimicrobial effector mechanism is the production of neutrophil extracellular traps (NETs), which are released during a programmed reactive oxygen species (ROS)-dependent cell death, the so-called NETosis. Although several functions of neutrophils have been analyzed under acidic conditions, the effect of extracellular acidosis on NETosis remains mainly unexplored and the available experimental results are contradictory. We performed a comprehensive study with the aim to elucidate the effect of extracellular acidosis on ROS-dependent NETosis of primary human neutrophils and to identify the underlying mechanisms. The study was performed in parallel in a CO_2_–bicabonate-buffered culture medium, which mimics *in vivo* conditions, and under HEPES-buffered conditions to verify the effect of pH independent of CO_2_ or bicarbonate. We could clearly show that extracellular acidosis (pH 6.5, 6.0, and 5.5) and intracellular acidification inhibit the release of ROS-dependent NETs upon stimulation of neutrophils with phorbol myristate acetate and immobilized immune complexes. Moreover, our findings suggest that the diminished NET release is a consequence of reduced ROS production and diminished glycolysis of neutrophils under acidic conditions. It was suggested previously that neutrophils can sense the border of inflamed tissue by the pH gradient and that a drop in pH serves as an indicator for the progress of inflammation. Following this hypothesis, our data indicate that an acidic inflammatory environment results in inhibition of extracellular operating effector mechanisms of neutrophils such as release of ROS and NETs. This way the release of toxic components and tissue damage can be avoided. However, we observed that major antimicrobial effector mechanisms such as phagocytosis and the killing of pathogens by neutrophils remain functional under acidic conditions.

## Introduction

Polymorphonuclear neutrophil granulocytes (PMN) are key players in the innate antimicrobial defense and in inflammatory responses. Beside phagocytosis, degranulation, production of reactive oxygen species (ROS), and secretion of chemoattractants, one important antimicrobial effector mechanism is the release of neutrophil extracellular traps (NETs) (1, 2). NETs are extracellular fibrous structures composed of chromatin, histones, and antimicrobial proteins, i.e., myeloperoxidase (MPO) (2). They are mostly released from activated neutrophils during the so-called NETosis, a programmed ROS-dependent cell death that is distinct from apoptosis and necrosis (3–6). NETosis is a multifactorial process, which requires ROS (3–5), distinct intracellular signaling pathways such as Akt, p38 MAPK, or MEK/ERK (7, 8), activation and/or translocation of enzymes [PAD4, MPO, and neutrophil elastase (NE)] (9–11), actin polymerization (11), and depends on glycolysis (12).

Although ROS and NETs contribute to pathogen containment, they are also double-edge swords as they can cause endothelial and tissue damage and thus contribute to inflammation and can lead to severe pathogenesis (13–20). Therefore, to prevent tissue damage, a proper regulation of neutrophil effector mechanisms is essential at sites of infection and inflammation. One of the factors, which can modulate neutrophil functions is the pH value of the inflammatory environment.

While the extracellular pH in the blood is firmly sustained at 7.35–7.45, inflammatory conditions are associated with acidification with pH values ranging from 5.5 to 7.0 (21–23). Acidic microenvironments are also described at sites of autoimmune inflammation such as the synovial fluid from joints of patients with rheumatoid arthritis (pH 6.0–7.0) (24) or in solid malignant tumors (pH 5.8–7.4) (25–27). Acidification in areas of inflammation is caused on the one hand by the massive infiltration and the intense metabolic activity of the immune cells. Due to their high energy demand and due to hypoxia at sites of inflammation, neutrophils switch their metabolism to anaerobic glycolysis, which leads to a local accumulation of lactic acid (28–30). On the other hand, activated neutrophils and macrophages produce superoxide by the NADPH oxidase (NOX2). This reaction in turn leads to the intracellular accumulation of protons and results in a transient intracellular acidification (31, 32). To maintain a physiological intracellular pH (pHi), the intracellular protons are transported out of neutrophils by Na^+^/H^+^ exchangers (NHE), H^+^ channels, $\text{Cl}/{\text{HCO}}_{3}^{-}$ exchangers, and the vacuolar type H-ATPase (V-ATPase) (31–33) and thus contribute to extracellular acidification. In addition to metabolic activity and ROS production of leukocytes, acidosis at sites of infection is also caused by short chain fatty acids (i.e., butyrate, acetate, and propionate) released as byproducts of the bacterial metabolism (34–37).

It has been reported that a change of the extracellular pH (pHo) delays neutrophil apoptosis (38, 39) and influences the cellular immune and bactericidal response (40). Most previous investigations observed inhibitory effects of extracellular acidosis on neutrophil functions, such as chemotaxis, bacterial killing, and superoxide production (39–43). However, activating effects of extracellular acidosis on neutrophils have also been described, such as calcium-mobilization, upregulation of the β2-integrin CD18, MPO release, and enhanced ROS production (H_2_O_2_) (38, 44). It was postulated by Trevani et al. that activating effects of extracellular acidosis on human neutrophils are dependent on the presence of extracellular bicarbonate and that use of different buffer systems *N*-2-hydroxyethylpiperazine-*N*′-2-ethanesulfonic-acid (HEPES) and cultivation conditions results in different effects on neutrophils (38). Thus, previous reports are contradictory.

Only few previous studies dealt with the influence of acidosis on NETosis/NET formation. In one study, dealing with the effect of anti-inflammatory drugs on NET-formation, phorbol myristate acetate (PMA)-induced NET release was shown to be enhanced under acidic conditions (pH 7.0 and 6.5) (45). In contrary, a recent study showed that the CO_2_ to bicarbonate ratio, which determines the pH, modulates the spontaneous and induced NET formation, suggesting that an acidic environment impairs NET formation (46). In another study, bovine neutrophils were treated with the fatty acid β-hydroxybutyrate (BHBA) (without investigating the pH values) and an inhibitory effect on NET formation and NET bactericidal activity was observed (47). Although, in the latter study, the pH values were not determined, both above mentioned studies suggest that acidosis or acidic components such as fatty acids can modulate NETosis.

The aim of this study was to investigate whether extracellular acidosis can modulate ROS-dependent NETosis of primary human neutrophils and to identify underlying mechanisms. In our comprehensive study, we investigated the effects of acidic pH on PMA- and immobilized immune complex (iIC)-induced NETosis in a ${\text{CO}}_{2}-{\text{NaHCO}}_{3}^{-}$-buffered medium system compared to HEPES-buffered conditions. Both, PMA- and iIC-induced NET release depends on the formation of ROS by NOX2 and MPO (3, 4, 6, 8). In addition to ROS and NETs, further neutrophil functions such as oxidative burst, phagocytosis, apoptosis, and bacterial killing were analyzed under acidic conditions. We could clearly show that extracellular acidosis inhibits the production of ROS and the release of NETs by primary human neutrophils in both systems while the apoptosis was delayed, the phagocytic capacity was stable and the bacterial killing was enhanced under extracellular acidosis.

## Materials and Methods

### Ethics Statement

Blood collection was conducted with the understanding and written consent of each participant and was approved by the Ethical Committee of the Medical Faculty of the University of Lübeck (05-124).

### Isolation of Primary Human Neutrophils

Peripheral heparinized blood was collected by venipuncture from healthy adult volunteers. Neutrophils were isolated as described previously using Percoll gradient centrifugation (48). The purity of granulocytes was >99% as determined by morphological examination of Giemsa-stained cytocentrifuge slides. The viability of the cells was >99% as determined by trypan blue exclusion.

### Media Acidification and Cell Culture

In this study, HEPES-buffered (Biochrom, Germany) or bicarbonate-buffered RPMI 1640 medium (Sigma-Aldrich, Germany) supplemented with 0.5% human serum albumin (HSA, Baxter, Germany) and 4 mM l-glutamin (Biochrom) was used. As physiological pH, we used pH 7.4. Acidification of the media to pH 6.5, 6.0, or 5.5 was achieved by addition of isotonic HCl. The pH value was determined using a pH meter at 37°C. Extracellular acidification was achieved by resuspending cell pellets of freshly isolated neutrophils in media previously adjusted to the desired pH values. Cells were cultured at 37°C. For the bicarbonate system, humidified 10% CO_2_ atmosphere, which mimics respiratory acidosis, was used to maintain the acidic pH values. The formulation of RPMI 1640 medium was modified for pH 7.4 by increasing the NaHCO_3_ concentration from 2.2 to 3.2 g/l to maintain pH 7.4 at 10% CO_2_. Following the Henderson–Hasselbach equation, the buffer range of the used CO_2_–bicarbonate-buffered medium system is from pH 5.1 to 7.4 (${\text{HCO}}_{3}^{-}$ pK_s_ = 6.1 at 37°C). Some experiments were performed under CO_2_ free conditions with HEPES-buffered RPMI 1640 medium with 20 mM HEPES (buffer range 6.8–8.2; pK_s_ = 7.39 at 37°C) or with double buffered RPMI 1640 medium (2 g/l NaHCO_3_, 10 mM HEPES) under 5% CO_2_. Neutrophils were preincubated for 30 min in the desired medium before running an assay.

### Assessment of Neutrophil Viability

The viability of neutrophils was analyzed by flow cytometry using Annexin V-FITC (Promokine, Germany) and propidium iodide (PI) (Sigma-Aldrich) staining according to the manufacturers’ instruction. Cells were analyzed by flow cytometry using a FACS CantoII flow cytometer and Diva software (BD Biosciences, USA).

### Phagocytosis Assay

Neutrophils (5 × 10^5^ cells/100 μl) were preincubated for 30 min in bicarbonate- or HEPES-buffered medium (pH 7.4, 6.5, 6.0, and 5.5). Subsequently, Alexa-Fluor 488 conjugated opsonized non-viable *Staphylococcus aureus* bioparticles (Invitrogen; *S. aureus* to neutrophil ratio 2:1) or FluoSphere carboxylate-modified latex microspheres with a diameter of 1 µM [Invitrogen; final concentration of 0.015% (v/v)] were added and the co-culture was incubated for further 30 min. Cultures were placed on ice to stop phagocytosis, cells were washed to remove extracellular bacteria/beads, and trypan blue was added to quench fluorescence of extracellular bacteria/beads sticking on the neutrophil surface. Phagocytosis was assessed by flow cytometry using a FACS CantoII flow cytometer.

### Bacterial Killing Assay

A bacterial killing assay with human neutrophils and opsonized *S. aureus* (ATCC 25923) was performed as previously described (49). Shortly, 10 × 10^6^ neutrophils per milliliter were incubated for 30 min in HEPES- or bicarbonate-buffered medium with different pH values. Also, 9 × 10^5^ PMN were then co-incubated for 30 min at 37°C with 9 × 10^6^ opsonized bacteria under different pH values in bicarbonate- or HEPES-buffered media. Following co-incubation, cells were lysed and the bacterial growth/survival was measured in a Tecan infinite M200 Pro reader (Tecan) on basis of changes in optical density (OD). Series of 1:2 dilutions from the stock bacterial suspension were measured in parallel and used to calculate the percentage of bacterial survival. The different pH values did not impair the viability and did not affect the growth kinetics of *S. aureus* (data not shown).

### Neutrophil Stimulation with Immobilized IC or PMA

Reactive oxygen species and NET studies were performed with 20 nM PMA or plate-bound iIC. PMA is the most widely used inducer of ROS-dependent NETosis (50). iIC play a role in autoimmune diseases and are also known to induce ROS-dependent NET release (8). iIC were formed by using HSA and rabbit polyclonal anti-HSA-IgG (Sigma-Aldrich, Germany), as described previously (8, 51). For functional assays, neutrophils in appropriate assay medium were transferred to iIC-coated wells. Uncoated wells were used for medium control and PMA stimulation.

### Inhibitor Studies

In some studies, neutrophils were preincubated for 30 min at 37°C with various inhibitors in assay medium before running the assay. For analysis of signaling pathways, 10 µM UO126 (ERK inihibitor, Cell Signaling Technology, USA), 10 µM VIII (Akt inhibitor, Calbiochem, Germany), or 10 µM SB 203580 (p38 MAPK inhibitor, Calbiochem) were used. To inhibit the Na^+^/H^+^ exchanger (NHE), neutrophils were preincubated with 10 µM of the NHE-inhibitor DMA [5-(*N*,*N*-dimethyl)amiloride hypochloride, Sigma]. The H^+^ATPase (V-ATPase) was inhibited by adding 100 nM Bafilomycin A1 (InvivoGen, USA). The glycolysis pathway was inhibited by use of 10 mM of the hexokinase-inhibitor 2-deoxyglucose (2DG, Sigma) or 100 µM of the glyceraldehyse-3-phosphate-dehydrogenase inhibitor sodium idoacetate (SIA, Sigma). Neutrophils exposed to the appropriate solvent [dimethyl sulfoxide (DMSO)] concentrations and untreated neutrophils served as controls. All inhibitors were freshly prepared for each experiment. The used inhibitors and the final DMSO concentration (0.1% v/v) had no toxic effects on neutrophils within 7 h, as analyzed by the Annexin V-/PI staining (data not shown).

### NET Assays

Neutrophil extracellular trap formation of neutrophils was quantified in culture by using the fluorescence-based SYTOXgreen real-time assay and in supernatants by a capture ELISA that detects MPO-associated DNA. For visualization of NETs, fluorescence microscopy (FM) and scanning electron microscopy (SM) were performed.

### SYTOXgreen Kinetic Assay

The time kinetics of NET release was assessed by using the non-cell permeable dsDNA dye SYTOXgreen (Invitrogen) (1, 4, 52). To exclude a direct influence of pH on SYTOXgreen, a control experiment with ds Lambda DNA was carried out. No difference in SYTOXgreen fluorescence intensity between pH 7.4, 6.5, 6.0 and 5.5 in HEPES- and bicarbonate-buffered medium was observed (data not shown). This was also shown by others (46). For NET kinetics, 10^6^ neutrophils per milliliter were incubated at 37°C in iIC-coated or uncoated (medium control, PMA stimulation) FLUOTRAC™ 600 plates (Greiner Bio-One) in bicarbonate- or HEPES-buffered medium with different pH values containing 5 µM SYTOXgreen. The NET-bound SYTOXgreen fluorescence (excitation: 488 nm, emission: 510 nm) was analyzed for 7 h every 5 min at 37°C by using Tecan infinite M200 Pro reader and Tecan i-control 1.7 Software. For bicarbonate-buffered conditions, CO_2_ control was achieved during the assay by the use of a Tecan gas module. For statistical analysis, the area under the curve (AUC) was calculated (for PMA stimulation, from 4 h, for iIC stimulation, from 7 h).

#### MPO–DNA Complex (NET) ELISA

Since NETs contain both DNA and MPO, a MPO–DNA complex ELISA was used to detect and quantify soluble NETs in culture supernatants as previously described (53–55). Briefly, 96-well ELISA maxisorp plates (Nunc) were coated with 5 µg/ml mouse anti-human MPO (BioRad) over night at 4°C. After three washing steps and blocking with 1% BSA, 20 µl of cell culture supernatant (from 1 × 10^6^ neutrophils per milliliter) together with 80 µl incubation buffer and 4 µl peroxidase labeled anti DNA mAB (both from Cell Death Detection ELISA Plus, Sigma) was added to the wells. Following 2 h incubation, the wells were washed once and 100 µl peroxidase substrate was added. The OD at 405 nm was measured after 20 min in an ELISA reader (Tecan). Measured OD values of soluble MPO–DNA complexes were normalized to the respective pH blank control (bicarbonate or HEPES medium without PMN) and are depicted as increase of NET formation.

#### Microscopical Assessment of NETs

Fluorescence microscopy and scanning electron microscopy was used for visualization of NETs. For FM, 10^6^ neutrophils per milliter were incubated in iIC-coated ibiTreat μ-slides (ibidi) for 7 h when iIC was used as stimulus or for 4 h in Poly-Ly-Lysin coated μ-slides when PMA was used as stimulus. Following fixation with 4% paraformaldehyde (Sigma-Aldrich), staining of MPO and DNA by using mouse anti-human MPO antibody (1:500, AbD Serotec, Germany) and SYTOXgreen was carried out as described previously (4, 56). Samples were analyzed with the AxioVert A.1 using the the Axiocam HRc and Axio Vision Rel. 4.8 software (all Carl Zeiss, Germany) or with the Keyence BZ-9000E using the BZ II Analyzer Software (KEYENCE, Germany).

For scanning electron microscopy, neutrophils (10^6^ per milliliter) were settled on iIC-coated or uncoated (medium control, PMA) thermanox coverslips (Greiner BioOne). Following incubation for 7 h (iIC)/4 h (PMA), the supernatant was removed and samples were fixed with 1 ml Monti-Graziadei solution and processed for SM as described (56). Preparates were examined with a Zeiss EVO HD 15 (Zeiss).

### ROS Assays

Three different methods were used to detect ROS production by human neutrophils, including a fluorescent probe assay and chemiluminescence assays.

#### Detection of Intra- and Extracellular ROS

The luminol-based chemiluminescence assay was used to detect the sum of intra- and extracellular ROS, mainly MPO-generated metabolites such as hydroxyl radicals (57, 58). This assay was previously shown to be functional under acidic conditions (39). Neutrophils (2 × 10^6^/ml) in bicarbonate- or HEPES-buffered medium containing 60 µM luminol (Sigma-Aldrich) were transferred to iIC-coated or uncoated (PMA, medium control) 96-well LUMITRAC™600 plates (Greiner Bio-One) and ROS-dependent chemiluminescence was analyzed using an infinite 200 reader and the Tecan i-control 1.7 Software (Tecan, Germany). ROS release was monitored for 1 h every 2 min at 37°C. For statistical analysis, the AUC of each sample was calculated.

#### Detection of Extracellular Superoxide

Extracellular superoxide was detected by using the lucigenin-amplified chemiluminescence assay (56, 58). This assay was performed the same way as the luminol assay, but with 0.2 mM lucigenin (Alexis, Germany) instead of luminol.

#### Detection of Intracellular ROS

The intracellular ROS production of individual cells was measured by flow cytometry using the substrate dihydrorhodamine 123 (DHR 123, Invitrogen) that is oxidized by ROS to the fluorescent rhodamine 123. By using DHR 123 hydrogen peroxide but also superoxide anions can be detected intracellularly (59). Neutrophils (2 × 10^6^/ml) were loaded with 2 µM DHR and were then cultivated under physiological versus acidic conditions (unstimulated, PMA-, or iIC stimulated). Following 30 min incubation, the reaction was stopped on ice and fluorescence intensity of rhodamine 123 was immediately analyzed by FACS (Canto II).

### Glucose Uptake Assay

To analyze the uptake of glucose, the fluorescent glucose analog 2(*N*-(7-nitrobenzen-2oxa-1,3-diazol-4-yl)amino)-2-deoxyglucose (2-NBDG) (Cayman) was used. Also, 5 × 10^5^ neutrophils (5 × 1 0^6^/ml) were preincubated for 30 min in glucose-free XF-assay medium (SeahorseBiosciences, Denmark) supplemented with NaHCO_3_ (2 g/l) or 20 nM HEPES at pH 7.4 or pH 6.0 and were then stimulated for 30 min with 20 nM PMA or left untreated. And, 10 min before the end of stimulation time, 100 mg/ml 2-NBDG was added. Following centrifugation and washing, cells were suspendend in FACS buffer and the uptake of 2-NBDG was analyzed by flow cytometry.

### Lactate Assay

A lactate assay kit (Sigma-Aldrich) was used to detect the metabolic compound L(+)-Lactate in supernatants of neutrophil cultures. Neutrophils (5 × 10^6^/ml) were preincubated for 30 min in bicarbonate-buffered RPMI medium (without HSA or FCS) at pH 7.4 and 6.0 and were then stimulated for 90 min with 20 nM PMA, iIC, or 100 ng/ml LPS or left untreated. Following incubation at 37°C under 10% CO_2_ atmosphere, the supernatants of the cultures were filtered through 10 kDa molecular weight cutoff ultracentrifuge filters (Amicon) to remove lactate dehydrogenase, and 5 µl of the filtrate was directly used in the lactate assay kit following manufactures instruction. For calculation of lactate concentration, the resulting OD data were corrected for the background by subtracting the appropriate blank value (medium pH 7.4/pH 6.0—the OD values were the same) and the concentration (nanograms per microliters) of lactate was calculated by interpolation from standard curve.

### Western Blot Analysis

Neutrophils (5 × 10^6^/ml) were left unstimulated or were stimulated with iIC/PMA for 15 min at 37°C in medium with different pH values. In some experiments, neutrophils were preincubated with various inhibitors. Whole cell lysates were prepared using TCA as described (60). Western blot analysis was carried out by using antibodies against human phospho Akt (Thr308), phospho-p44/42 MAPK (ERK1/2, thr202/Tyr204), phospho p38 MAPK (Thr180/Tyr182), or beta-actin (all from Cell Signaling Technology) and probed with HRP-conjugated anti-rabbit or anti-mouse IgG (New England Biolabs, USA). The signals were detected by using Immobilon Western Chemiluminescence HRP substrate (Millipore, USA) by using the Fusion Fxt Chemiluminescence reader (Vilber Loumat, Germany). Signals of pAkt, pERK1/2, or pp38 were normalized to beta-actin signals on the same blots or of the same sample by using ImageJ software (NIH, USA) (61).

### Statistical Analysis

If not stated differently, the presented data were collected/generated from minimum of three independent experiments with neutrophils isolated from different blood donors. Statistical analysis was performed with the GraphPad Prism software 6 using the one-way ANOVA followed by Bonferroni *t*-test for multiple comparisons. A *p*-value ≤0.05 was considered statistically significant.

## Results

### Methodological Considerations to the Applied Media and Buffer Systems

To perform the experiments as close as possible to conditions of *in vivo* extracellular environment a ${\text{CO}}_{\text{2}}-{\text{HCO}}_{3}^{-}$-buffered medium system was used (${\text{HCO}}_{3}^{-}$ pK_s_ = 6.1 at 37°C). To maintain acidic pH values, it was necessary to modulate the bicarbonate and/or the CO_2_ concentration. We consistently used 10% CO_2_, which mimics a respiratory acidosis. To maintain a physiological pH of 7.4 under the high CO_2_ level, the formulation of RPMI-1640 medium was modified for pH 7.4 by increasing the NaHCO_3_ concentration from 2.2 to 3.2 g/l. Under these ${\text{CO}}_{\text{2}}-{\text{HCO}}_{3}^{-}$ conditions, the adjusted pH values (7.4, 6.5, 6.0, and 5.5) were stable for 7 h, which is the maximal duration of performed NETs experiments. Interestingly, we observed that NaHCO_3_ (2.2 g/l)-buffered medium induces a NET signal by primary human neutrophils without an additional stimulus at room air CO_2_ level (Figure S1 in Supplementary Material). The presence of 5 and 10% CO_2_ inhibits the NaHCO_3_-induced NET signal (Figure S1 in Supplementary Material). Thus, the effects of bicarbonate and CO_2_ on NET release had to be taken into consideration when using bicarbonate-buffered media, especially when different bicarbonate or CO_2_ levels were used to maintain distinct pH values. Therefore, to verify CO_2_/bicarbonate-independent effects of pH on neutrophil functions, we used HEPES-buffered medium in parallel samples. HEPES (pK_s_ = 7.39 at 37°C) is a zwitterionic single component buffer that works independent of CO_2_. HEPES-buffered medium does not induce a NET-signal by human neutrophils, neither under room air nor in an atmosphere containing 10% CO_2_ (Figure S1 in Supplementary Material).

### Extracellular Acidosis Delays Neutrophil Apoptosis

**Figure 1:**
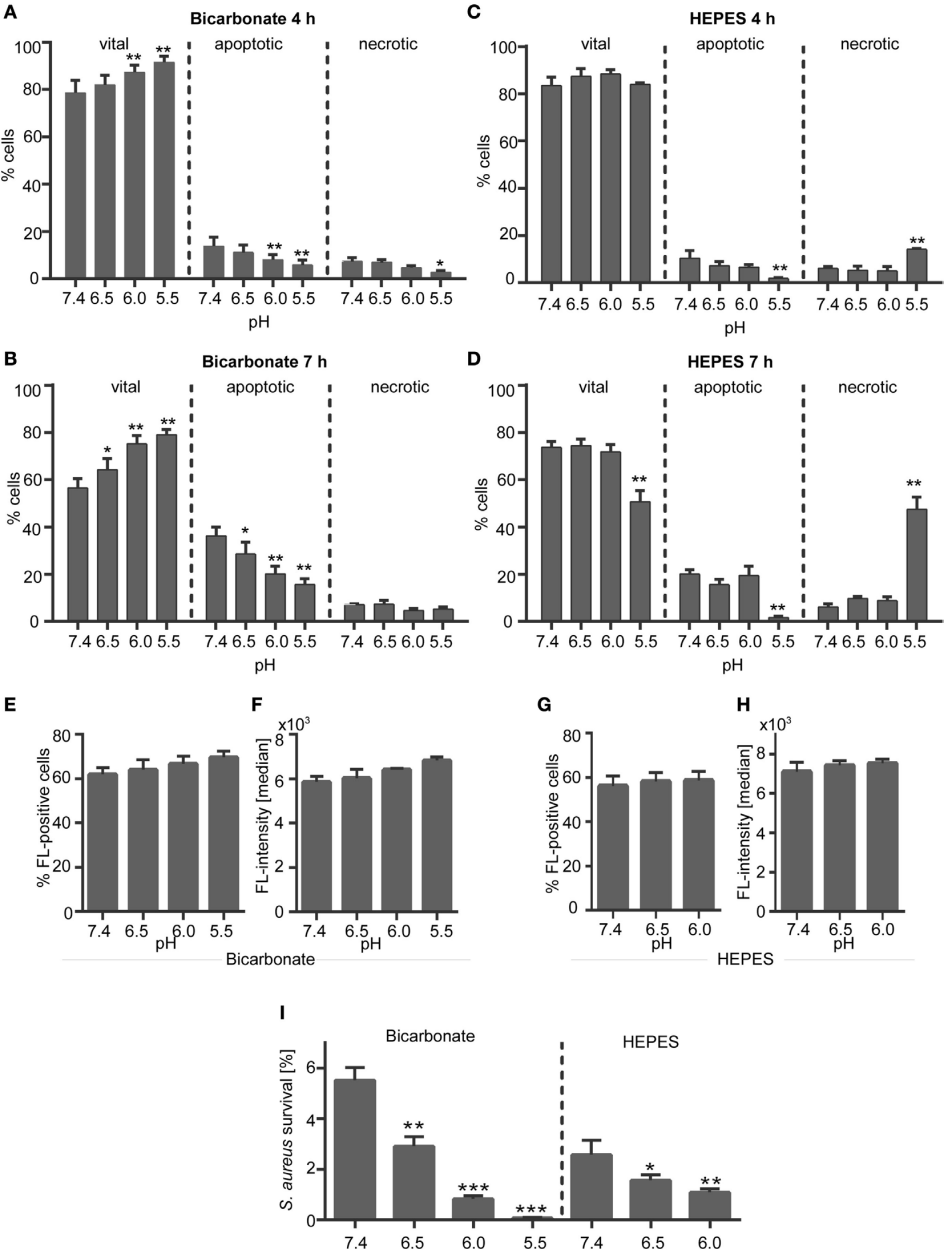
**Effect of extracellular acidosis on neutrophil survival and phagocytosis**. **(A–D)** Neutrophils (10^6^ cells/ml) were incubated for 4 or 7 h in **(A,B)** bicarbonate (10% CO_2_) or **(C,D)** *N*-2-hydroxyethylpiperazine-*N*′-2-ethanesulfonic-acid (HEPES)-buffered RPMI 1640 at pH 7.4, 6.5, 6.0, and 5.5. Apoptosis and necrosis rate of neutrophils was then analyzed by flow cytometry using Annexin V-FITC and propidium iodide staining. Mean values ± SEM (% cells) from three independent experiments for each pH value are given. *p < 0.05, **p < 0.01 as compared to pH 7.4 **(E–H)** neutrophils (5 × 10^5^cells/100 μl) were preincubated for 30 min in bicarbonate- or HEPES-buffered medium at pH 7.4, 6.5, 6.0, 5.5 and were then co-incubated for 30 min with opsonized *Staphylococcus aureus* bioparticles. Phagocytosis of bioparticles was assessed by flow cytometry. **(E,G)** The percentage of neutrophils that phagocytosed *S. aureus* bioparticles (mean ± SEM) and **(F,H)** the mean fluorescence per cell (median ± SEM, right panel) are shown (*n* = 3). **(I)** 9 × 10^5^ neutrophils were co-incubated for 30 min with 9 × 10^6^ opsonized *S. aureus* in bicarbonate- or HEPES-buffered medium at pH 7.4, 6.5, 6.0, and 5.5. Following lysis of neutrophils, bacterial survival was measured on basis of changes in optical density. Percent of surviving bacteria (mean ± SEM, *n* = 3) are shown. *p < 0.05, **p < 0.01, and ***p < 0.001 as compared to pH 7.4.

To exclude an apoptosis- or necrosis-inducing effect of the media or acidosis on neutrophils, the survival of human neutrophils that were cultivated in bicarbonate- or HEPES-buffered media (pH 7.4, 6.5, 6.0, or 5.5) was analyzed after 4 and 7 h incubation. These time points were chosen, as these are the durations of PMA- (4 h) or iIC-stimulated (7 h) NET experiments. Extracellular acidosis significantly delayed apoptosis and prolonged survival of neutrophils as compared to pH 7.4 within 4 and 7 h in bicarbonate-buffered medium (Figures 1A,B). Necrosis was not significantly reduced, except at pH 5.5 after 4 h. A tendency toward a longer survival and less apoptosis under acidic conditions was also visible after 4 h incubation in HEPES-buffered medium (Figure 1C), but the effects were not significant, neither for 4 h (Figure 1C) nor for 7 h (Figure 1D) time points. Decreased neutrophil survival and apoptosis rate was observed at pH 5.5 due to an increase in necrosis within 4 and 7 h (Figures 1C,D). The necrosis-inducing effect of strong acidic conditions (pH 5.5) can be explained by the buffer range of the HEPES medium, which is from pH 6.8 to 8.2 meaning that there is no efficient buffering effect at pH 5.5 and thus necrosis of cells is induced. Based on these results in subsequent experiments, we used pH 7.2, 6.5, 6.0, and 5.5 for bicarbonate-buffered conditions, but only pH 7.4, 6.5, and 6.0 for HEPES-buffered conditions.

### Neutrophil Phagocytosis and Bacterial Killing Are Functional in an Acidic Environment

We analyzed the phagocytosis and bacterial killing capacity of neutrophils in order to check if the neutrophils are still functional under the chosen acidic environments. Phagocytosis was assessed by use of non-viable opsonized *S. aureus* bioparticles, which did not induce formation of NETs by primary human neutrophils (Figure S2 in Supplementary Material). The percentage of cells that phagocytosed *S. aureus* bioparticles (Figures 1E,G) and the phagocytic capacity (fluorescence/bacteria per cell) (Figures 1F,H) was stable and was not reduced under acidic conditions neither in the bicarbonate–CO_2_-buffered system (Figures 1E,F) nor under HEPES-buffered conditions (Figures 1G,H). The same results were observed for the phagocytosis of FluoSphere latex beads (Figure S3 in Supplementary Material) verifying that the phagocytic capacity of neutrophils is stable in an acidic environment. Co-incubation of viable opsonized *S. aureus* with human neutrophils resulted in efficient killing of bacteria (Figure 1I). And physiological pH of 7.4 less than 6% of bacteria survived under bicarbonate-buffered conditions and less than 3% of bacteria under HEPES-buffered conditions (Figure 1I). Increasing acidosis significantly enhanced the bacterial killing capacity of neutrophils resulting in a decreased survival of *S. aureus* under both HEPES and bicarbonate-buffered conditions (Figure 1I). The different pH values did not impair the viability and growth of *S. aureus* when neutrophils were not present (data not shown). These data indicate that the neutrophils are still functional under acidic conditions used in this study.

### NET Formation Is Inducible under Both Bicarbonate- and HEPES-Buffered Conditions

**Figure 2:**
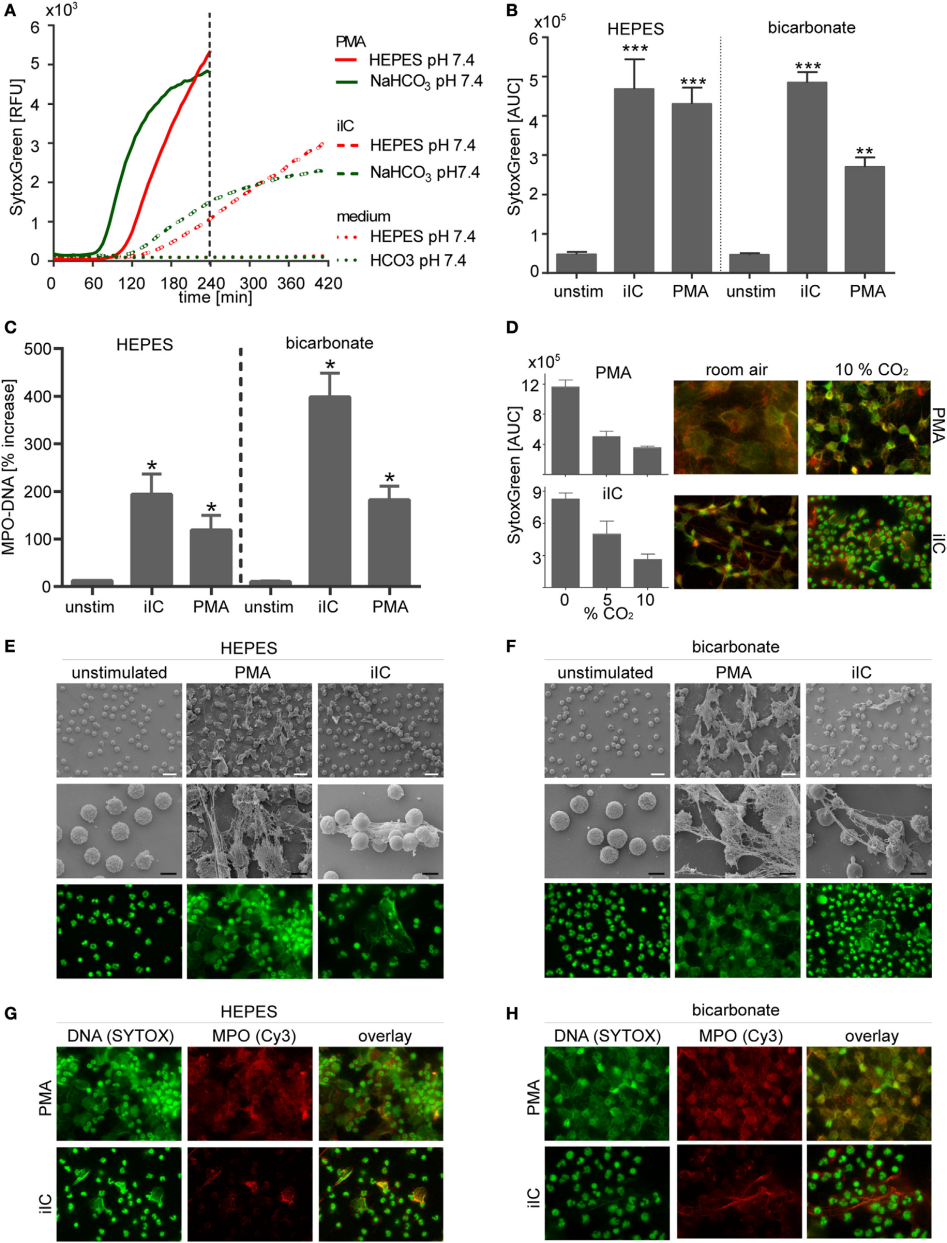
**Neutrophil extracellular traps are induced by phorbol myristate acetate (PMA) and immobilized immune complex (iIC) under *N*-2-hydroxyethylpiperazine-*N*′-2-ethanesulfonic-acid (HEPES)- and bicarbonate-buffered conditions at pH 7.4**. A total of 10^6^ neutrophils/ml were preincubated for 30 min under bicarbonate- or HEPES-buffered conditions at pH 7.4 and were then stimulated with PMA, iIC, or left untreated. Release of neutrophil extracellular traps (NETs) was monitored for 4 h (PMA) or 7 h (iIC) at 37°C (under 10% CO_2_ for bicarbonate-buffered system). **(A)** Representative real-time kinetics of NET release measured by staining with SYTOXgreen and **(B)** area under the curve (AUC) values (mean ± SEM) of NET-dependent relative fluorescence intensities (RFUs) as measured by the SYTOXgreen assay. *n* = 3–9, **p < 0.01, ***p < 0.001 as compared to unstimulated samples. **(C)** Myeloperoxidase (MPO)–DNA ELISA was used to measure soluble NETs in neutrophil supernatants. Data (mean ± SEM) are normalized to the respective pH blank controls (bicarbonate or HEPES medium without cells) and depicted as percentage increase. *n* = 3, *p < 0.05 as compared to unstimulated. **(D)** CO_2_ modulates PMA- and iIC-induced NETosis under bicarbonate-buffered conditions. SYTOXgreen AUC values (mean ± SEM) and fluorescence microscopy (FM) images (DNA green MPO red, overlay orange/yellow) of PMA (20 nM, 4 h) or iIC (7 h)-stimulated neutrophils incubated under room air, 5% CO_2_, or 10% CO_2_ are shown. **(E,F)** Representative scanning electron microscopy (SM) and FM images of PMA- and iIC-induced NETs under **(E)** HEPES and **(F)** bicarbonate-buffered conditions. For FL microscopy, cells were fixed and stained for DNA by using SYTOXgreen (green). **(G,H)** Immunohistochemical staining of fixed PMA- and iIC-induced NETs with MPO (red) by using mouse anti-human MPO and Cy3 conjugated goat anti-mouse IgG. DNA was stained by SYTOXgreen (green). Overlay of green and red fluorescence images is shown. The white scale bar represents 20 µm and the black scale bar 10 µm.

**Figure 3:**
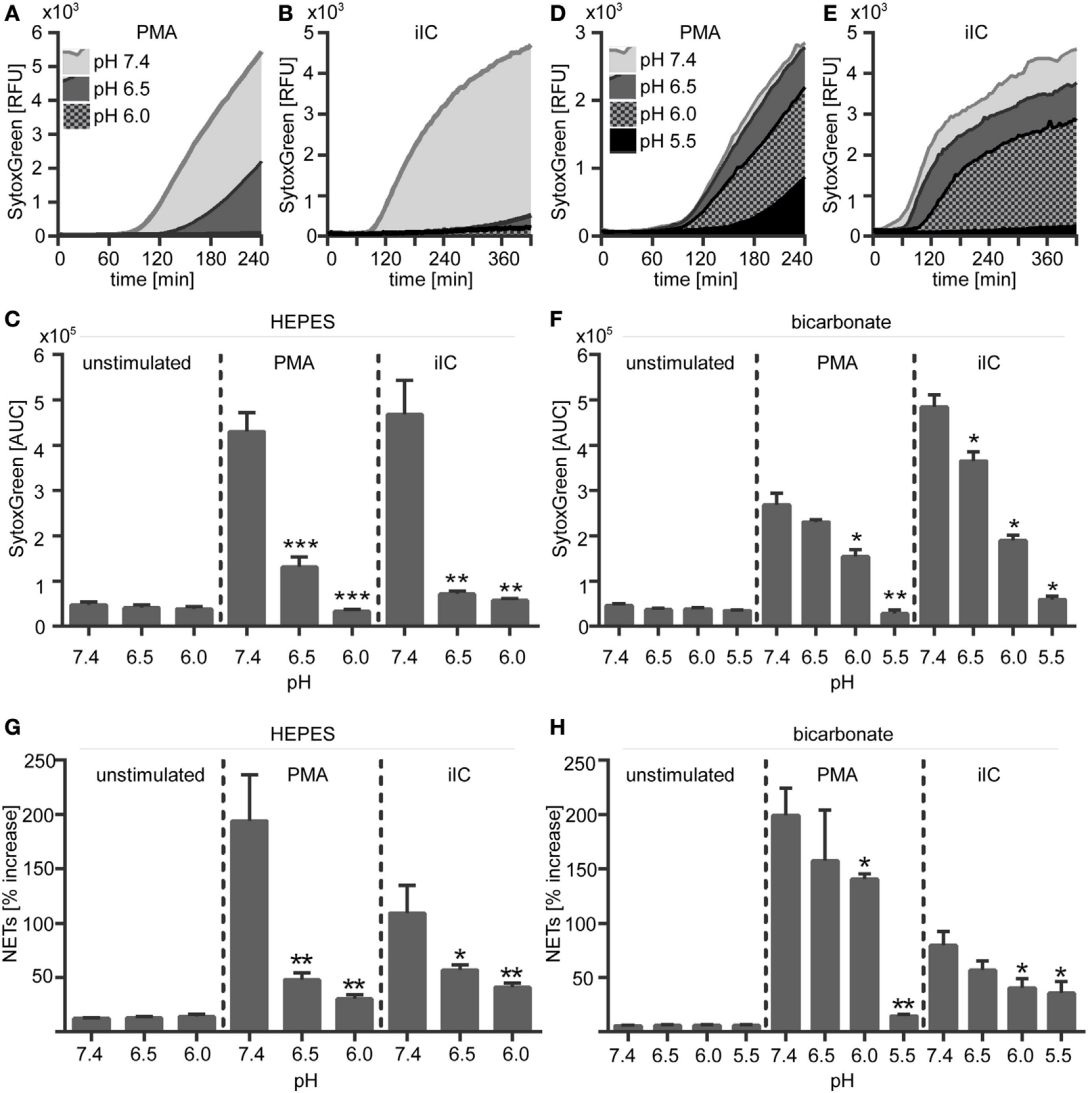
**Extracellular acidosis inhibits the release of phorbol myristate acetate (PMA)- and immobilized immune complex (iIC)-induced neutrophil extracellular trap (NETs) under *N*-2-hydroxyethylpiperazine-*N*′-2-ethanesulfonic-acid HEPES- and bicarbonate-buffered conditions**. 10^6^ neutrophils per milliliter were preincubated for 30 min under bicarbonate- or HEPES-buffered conditions at pH 7.4, 6.5, 6.0, and 5.5 and were then stimulated with PMA, iIC, or left untreated. Release of NETs was monitored for 4 h (PMA) or 7 h (iIC) by using the SYTOXgreen assay. **(A,B,D,E)** Representative real-time kinetics and **(C,F)** area under the curve (AUC) values (mean ± SEM) of NET-dependent relative fluorescence intensities (RFUs) as measured by the SYTOXgreen assay for **(A–C)** HEPES and **(D–E)** bicarbonate-buffered conditions. *n* = 3–9, **p < 0.01, ***p < 0.001 as compared to unstimulated samples. **(G,H)** Soluble myeloperoxidase–DNA complexes (NETs) were measured in neutrophil supernatants. Data (mean ± SEM) are normalized to the respective pH blank (bicarbonate or HEPES medium without cells) and depicted as percentage increase (*n* = 3, *p < 0.05, **p < 0.01 as compared to pH 7.4).

As shown in Figures 2A–C, both PMA and iIC significantly induced the release of NETs by human primary neutrophils at pH 7.4 under both bicarbonate- and HEPES-buffered conditions as detected by SYTOXgreen real-time kinetics and by MPO–DNA (NET) ELISA. Scanning electron and FM (Figures 2E,F) confirmed the SYTOXgreen and MPO–DNA assay results: PMA- and iIC-stimulated neutrophils released complex three-dimensional structures at pH 7.4, under both HEPES- and bicarbonate-buffered conditions. Double staining for DNA and MPO (Figures 3G,H) revealed that these structures consist of decondensed DNA (green) associated with antimicrobial proteins (MPO, red) (Figures 2G,H, overlay). The SYTOXgreen kinetics reveals that PMA- and iIC-induced NETs are released faster under bicarbonate- than under HEPES-buffered conditions (Figure 2A). This may be due to the presence of bicarbonate, as we observed that bicarbonate alone can induce a NET signal, which can be decreased/inhibited by CO_2_ (Figure S1 in Supplementary Material). Moreover, we found that an increase in CO_2_ concentration resulted in decreased PMA- and iIC-induced NETosis under bicarbonate-buffered conditions (Figure 2D). Especially, for iIC-induced NETosis, this effect was clearly obvious: while under room air, all cells released complex three-dimensional fibers composed of DNA and MPO (Figure 2D), only few NETs with a cloud like structure were released under 10% CO_2_ and most cells showed intact nuclear morphology. By using PMA, NET release was visible under both room air and CO_2_ conditions, but the NETs were bigger in size under room air as under CO_2_ conditions.

### Extracellular Acidosis Inhibits the Release of NETs

**Figure 4:**
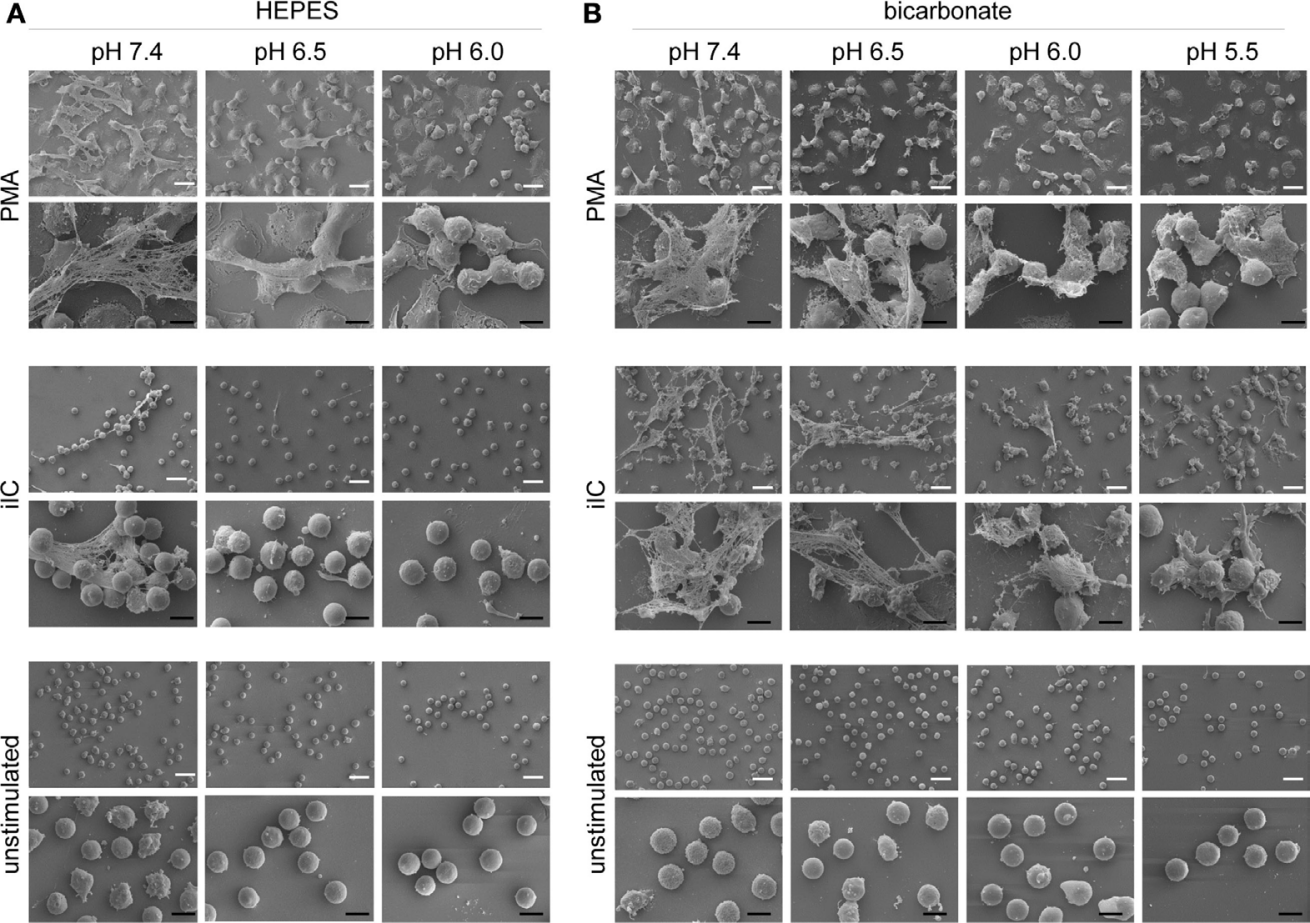
**Extracellular acidosis inhibits the release of neutrophil extracellular trap (NET) fibers**. Neutrophils (10^6^/ml) were allowed to settle on coverslips for 30 min under bicarbonate- or *N*-2-hydroxyethylpiperazine-*N*′-2-ethanesulfonic-acid (HEPES)-buffered conditions at pH 7.4, 6.5, 6.0, and 5.5 and were then stimulated with phorbol myristate acetate (PMA), immobilized immune complex (iIC), or left untreated. PMA-stimulated samples were fixed after 4 h and iIC-stimulated and unstimulated neutrophils after 7 h incubation time. Representative scanning electron images of PMA- and iIC-induced NETs under **(A)** HEPES and **(B)** bicarbonate-buffered conditions are shown. The white scale bar represents 20 µm and the black scale bar 10 µm.

We next assessed the effect of extracellular acidosis on the formation of NETs. SYTOXgreen kinetics (Figures 3A–F) and DNA–MPO complex ELISA (Figures 3G,H) revealed that increasing extracellular acidosis results in reduced iIC- and PMA-induced NET release under both bicarbonate- (Figures 3A–C,G) and HEPES-buffered conditions (Figures 3D–F,H). The inhibitory effect was stronger under HEPES-buffered conditions, where PMA-induced NET release was completely inhibited at pH 6.0 (Figure 3C). Under bicarbonate-buffered conditions, a statistically significant inhibitory effect was observed at pH 6.0 and 5.5 (Figure 3F). iIC-induced NET release was significantly inhibited at all tested acidic pH under both, HEPES, and bicarbonate-buffered conditions (Figures 3C,F). Extracellular acidosis alone, without an additional stimulus, did not induce NET formation by primary human neutrophils (Figure 3). The inhibitory effect of extracellular acidosis on PMA- and iIC-induced NET release was verified by scanning electron microscopy and revealed that increasing acidosis leads to less NETs and more intact cells (Figure 4). The inhibitory effect of extracellular acidosis on ROS-dependent NET formation was also visible under double buffered $\text{(HEPES}+{\text{HCO}}_{3}^{-})$ conditions (Figure S4 in Supplementary Material).

### Extracellular Acidosis Inhibits Neutrophil ROS Production

**Figure 5:**
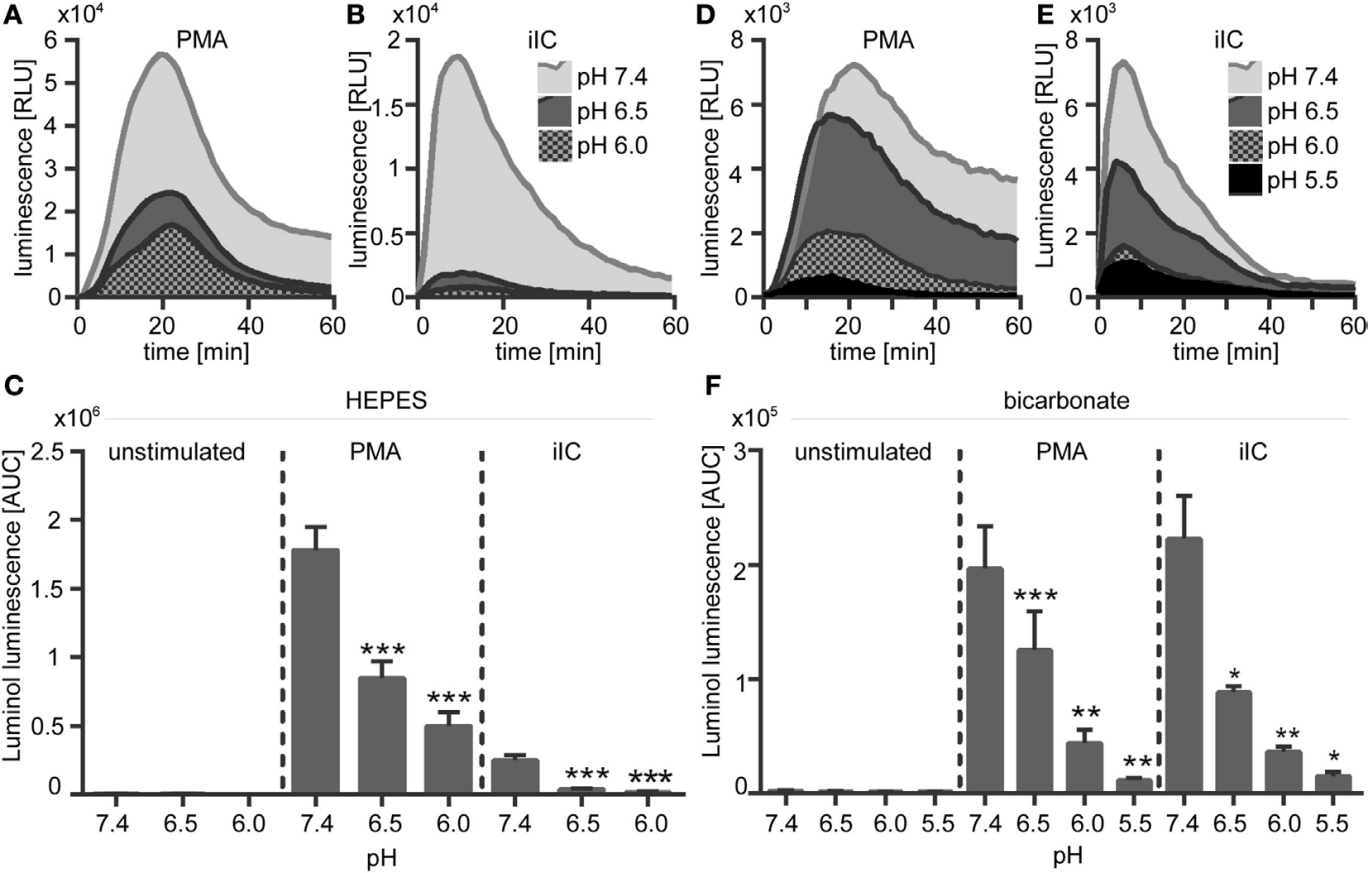
**Extracellular acidosis leads to decreased phorbol myristate acetate (PMA)- and immobilized immune complex (iIC)-induced reactive oxygen species (ROS) production in *N*-2-hydroxyethylpiperazine-*N*′-2-ethanesulfonic-acid (HEPES)- and bicarbonate-buffered media**. Neutrophils (2 × 10^6^/ml) were preincubated for 30 min under bicarbonate- or HEPES-buffered conditions at pH 7.4, 6.5, 6.0, and 5.5 and were then stimulated with PMA, iIC, or left untreated. Real-time analysis of intra- and extracellular myeloperoxidase-dependent ROS was monitored by using the luminol-assay for 1 h at 37°C (under CO_2_ for bicarbonate-buffered conditions). **(A,B,D,E)** Representative real-time kinetics and **(C,F)** area under the curve (AUC) values (mean ± SEM) of ROS-dependent chemiluminescence intensities (RLUs). *n* = 3–9 independent experiments (*p < 0.05, **p < 0.01, ***p < 0.001 as compared to pH 7.4).

**Figure 6:**
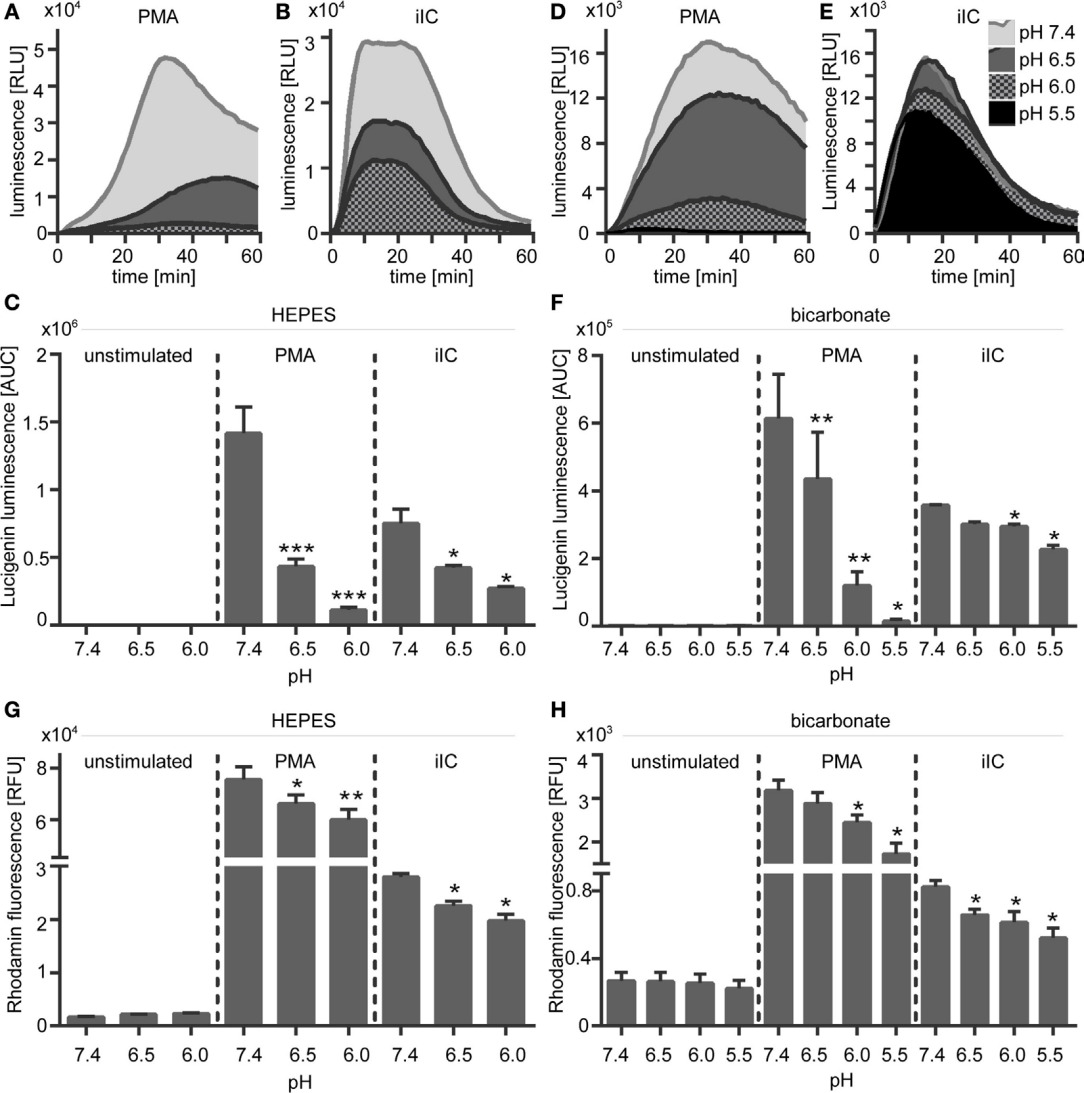
**Extracellular acidosis leads to decreased phorbol myristate acetate (PMA)- and immobilized immune complex (iIC)-induced superoxide production in *N*-2-hydroxyethylpiperazine-*N*′-2-ethanesulfonic-acid (HEPES)- and bicarbonate-buffered media**. Neutrophils (2 × 10^6^/ml) were preincubated for 30 min under bicarbonate- or HEPES-buffered conditions at pH 7.4, 6.5, 6.0, and 5.5 and were then stimulated with PMA, iIC, or left untreated. **(A–F)** Real-time analysis of extracellular superoxide was monitored by using the lucigenin assay for 1 h at 37°C (under CO_2_ for bicarbonate-buffered conditions). **(A,B,D,E)** Show representative real-time kinetics and **(C,F)** area under the curve (AUC) values (mean ± SEM) of reactive oxygen species (ROS)-dependent chemiluminescence intensities (RLU). *n* = 6 independent experiments. **(G,H)** Intracellular ROS production was analyzed by flow cytometry using DHR-123. Cells were preincubated for 30 min in bicarbonate- or HEPES-buffered medium and then stimulated for 30 min with PMA or iIC in the presence of DHR. Mean ± SEM (*n* = 3) values of detected rhodamin fluorescence are shown (*p < 0.05, **p < 0.01, and ***p < 0.001 as compared to pH 7.4).

The effect of extracellular acidosis on PMA- and iIC-induced ROS production of human neutrophils was assessed by using the luminol- and lucigenin-based chemiluminescence assays and the fluorescent probe DHR 123. Extracellular acidosis alone did not induce the production of ROS in unstimulated cells, as no signal was detected in the luminol, lucigenin, or in the DHR assay in unstimulated neutrophils under acidic conditions (Figures 5 and 6). Experiments using the chemiluminescence-based techniques revealed that extracellular acidosis results in decreased intra- and extracellular MPO-dependent ROS (Figure 5) and extracellular superoxide production (Figure 6) upon PMA and iIC stimulation as compared to ROS production at pH 7.4. The inhibitory effect was obvious for all tested acidic pH values under bicarbonate- and under HEPES-buffered conditions (Figures 5C,F and 6C,F). Extracellular acidosis also inhibited the intracellular ROS production, as measured by the DHR assay (Figures 6G,H). The effect was, however, more obvious under bicarbonate-buffered conditions. These results reveal an inhibitory effect of extracellular acidosis under both HEPES- and bicarbonate-buffered condition on PMA- and iIC-induced ROS production.

### Inhibition of the Na^+^/H^+^ Exchanger Decreases ROS and NET Production

As an approach to modulate pHi, PMA-stimulated neutrophils were treated with DMA, an inhibitor of the Na^+^/H^+^ exchanger NHE-1 or bafilomycin, an inhibitor of V-ATPase. Blocking of these proton transporters in activated neutrophils results in intracellular acidification (33).

**Figure 7:**
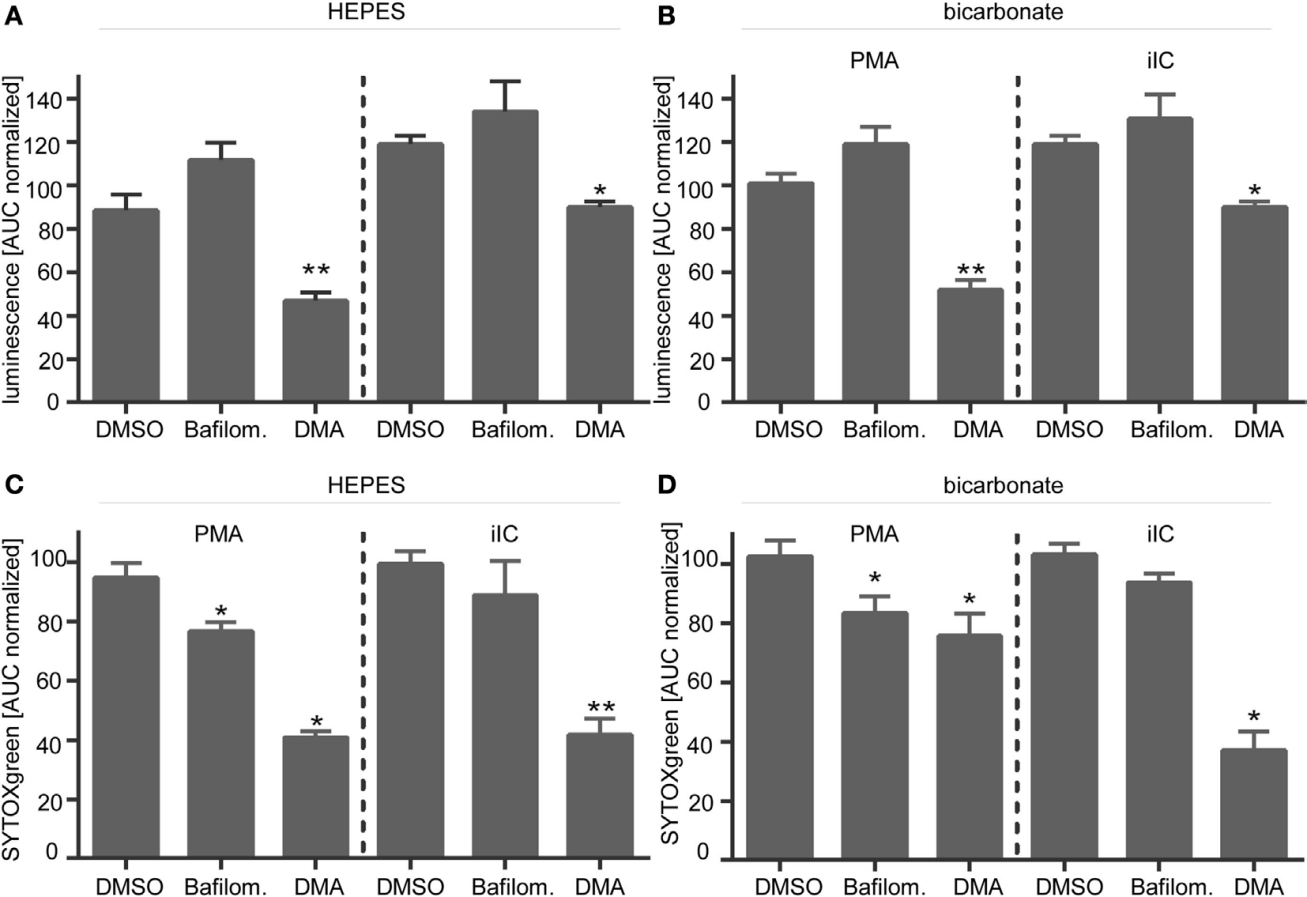
**Inhibition of NHE-1 leads to reduced phorbol myristate acetate (PMA)- and immobilized immune complex (iIC)-induced reactive oxygen species (ROS) and neutrophil extracellular traps (NETs)**. Neutrophils (2 × 10^6^/ml for ROS assays, 10^6^/ml for NET assay) were preincubated for 30 min under bicarbonate- or *N*-2-hydroxyethylpiperazine-*N*′-2-ethanesulfonic-acid-buffered conditions at pH 7.4 with inhibitors of NHE-1 (DMA, 10 µM), V-ATPase (Bafilomycin, 100 nM), solvent control [dimethyl sulfoxide (DMSO), 1:1,000], or left untreated (medium control) and were then stimulated with PMA or iIC. Release of ROS was monitored for 1 h and release of NETs for 4 h (PMA) or 7 h (iIC) at 37°C (under CO_2_ for bicarbonate-buffered system) by using the luminol- and the SYTOXgreen assays. **(A,B)** Normalized area under the curve (AUC) values (mean ± SEM) of ROS-dependent luminol-chemiluminescence and **(C,D)** of NET-dependent fluorescence intensities as measured by the SYTOXgreen assay. AUC values were normalized to PMA- or iIC-stimulated medium control (PMA/iIC stimulated neutrophils in medium pH 7.4 without solvent or inhibitors) *n* = 3, *p < 0.05, **p < 0.01 as compared to solvent control (DMSO).

Blocking of NHE-1 with DMA significantly reduced the ROS production (Figures 7A,B) and the NET release (Figures 7C,D) of PMA- and iIC-stimulated neutrophils under both bicarbonate- and HEPES-buffered conditions. The effects of the V-ATPase inhibitor bafilomycin were not so pronounced. We observed a significant reduced NET release upon PMA stimulation, but for iIC-stimulated neutrophils, the effects of bafilomycin were not significant (Figures 7C,D). The ROS signal was not significantly altered in bafilomycin-treated neutrophils (Figures 7A,B). Blocking of NHE-1 with DMA also results in reduced ROS and NET production of PMA-stimulated neutrophils under double-buffered conditions (HEPES + bicarbonate) (Figure S5 in Supplementary Material). As NHE-1 plays a major role for intracellular homeostasis and blocking of NHE-1 in activated neutrophils results in intracellular acidification, these results suggest that the ROS and NET production is inhibited by intracellular acidosis.

### Reduced ROS Are Responsible for Inhibition of NETosis under Extracellular Acidosis

**Figure 8:**
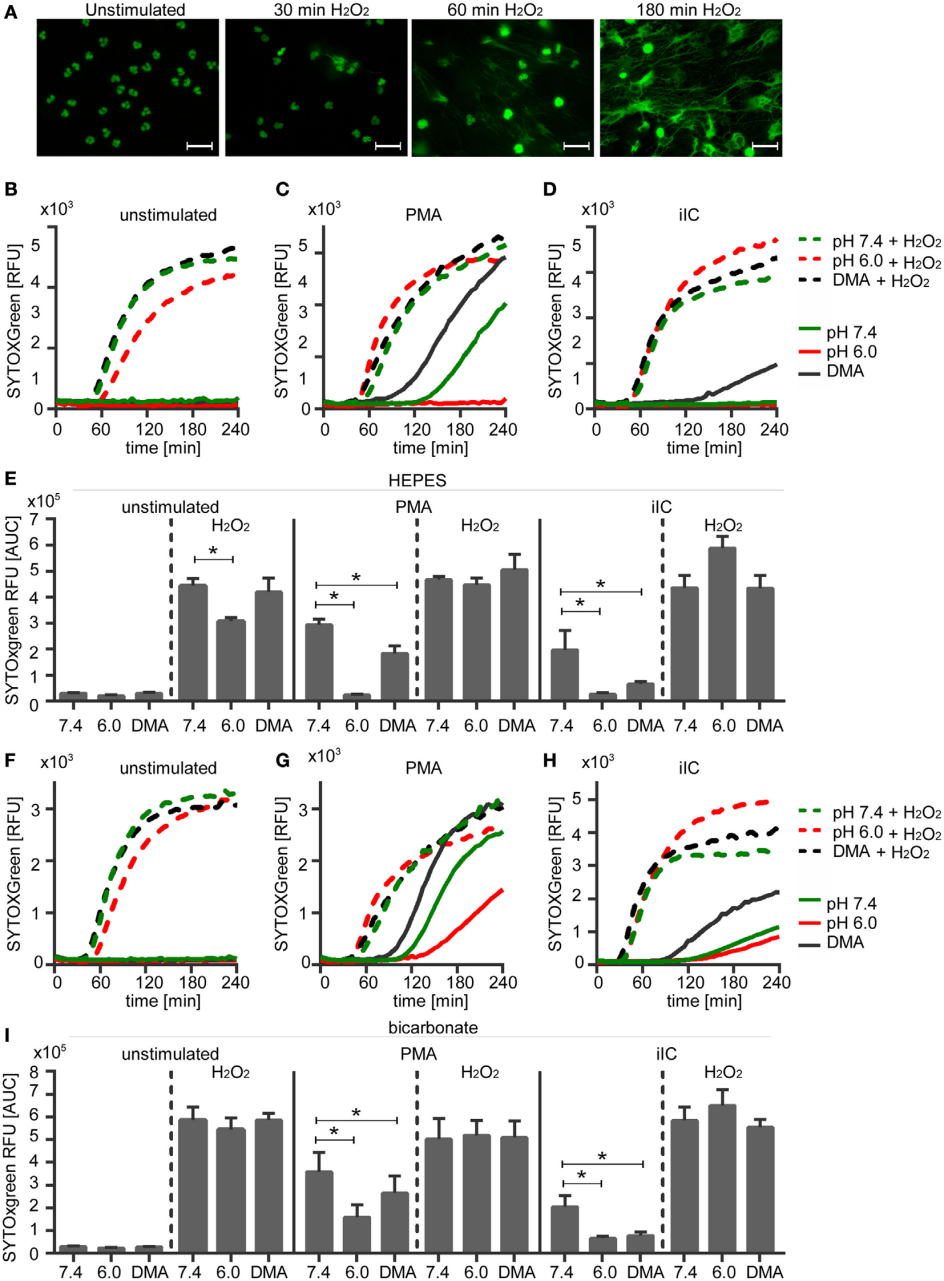
**Hydrogen peroxide treatment induces neutrophil extracellular traps (NETs) under physiological and acidic conditions**. 10^6^ neutrophils/ml were preincubated for 15 min under **(A,F–I)** bicarbonate or **(B–E)** *N*-2-hydroxyethylpiperazine-*N*′-2-ethanesulfonic-acid-buffered conditions at pH 7.4 (with or without 10 µM DMA) or 6.0 and were then stimulated with 20 nM phorbol myristate acetate (PMA), immobilized immune complex, or left untreated. Subsequently, 30 µM H_2_O_2_ was added and release of NETs was monitored for 4 h by using the SYTOXgreen assay. **(A)** Show representative fluorescence images of fixed, SYTOXgreen stained neutrophils treated with hydrogen peroxide for 30, 60, and 180 min. Scale bar represents 20 µM. **(B–D,F–H)** Representative real-time kinetics and **(E,I)** area under the curve (AUC) values (mean ± SEM, *n* = 3) of NET-dependent relative fluorescence intensities (RFUs) as measured by the SYTOXgreen assay are shown.

To check if the diminished ROS production under acidic conditions is the direct reason for inhibition of NETosis, unstimulated and PMA-stimulated neutrophils were treated with hydrogen peroxide, which directly induces NET formation. Upon treatment of unstimulated neutrophils with hydrogen peroxide, we observed first NET structures after 30 min and maximal NET release after 180 min (Figure 8A). NET release was monitored under extracellular physiological (pH 7.4) and acidic (pH 6.0) conditions and at acidic pHi (DMA). Treatment of neutrophils with hydrogen peroxide induced the release of NETs from unstimulated neutrophils and enhanced the NET release of PMA- or iIC-stimulated neutrophils under physiological conditions under both HEPES (Figures 8B,E) and bicarbonate-buffered conditions (Figures 8F,I). Hydrogen peroxide treatment also induced NET release from unstimulated neutrophils under acidic conditions (pH 6.0) and from cells with acidic pHi (DMA). In bicarbonate-buffered medium (Figures 8F,I), the NET release upon hydrogen peroxide treatment from unstimulated neutrophils was at the same level under pH 6.0 as under physiological pH 7.4. In HEPES-buffered medium (Figures 8B,E), hydrogen peroxide also induced a significant induction of NETs under extracellular acidosis, but not to the same level as under physiological pH 7.4. In both bicarbonate- and HEPES-buffered conditions, the acidosis-dependent inhibition of PMA- and iIC-induced NET formation was totally recovered by hydrogen peroxide to the same extent as under physiological conditions (Figures 8C–E,G–I). These data show that NETosis under acidic conditions can still occur when sufficient ROS are present and suggest that the diminished ROS production is the reason for reduced NET release under acidic conditions.

### Activation of Akt, ERK 1/2, or p38 MAPK Does Not Play a Role in the Acidosis-Dependent Inhibition of NETosis

**Figure 9:**
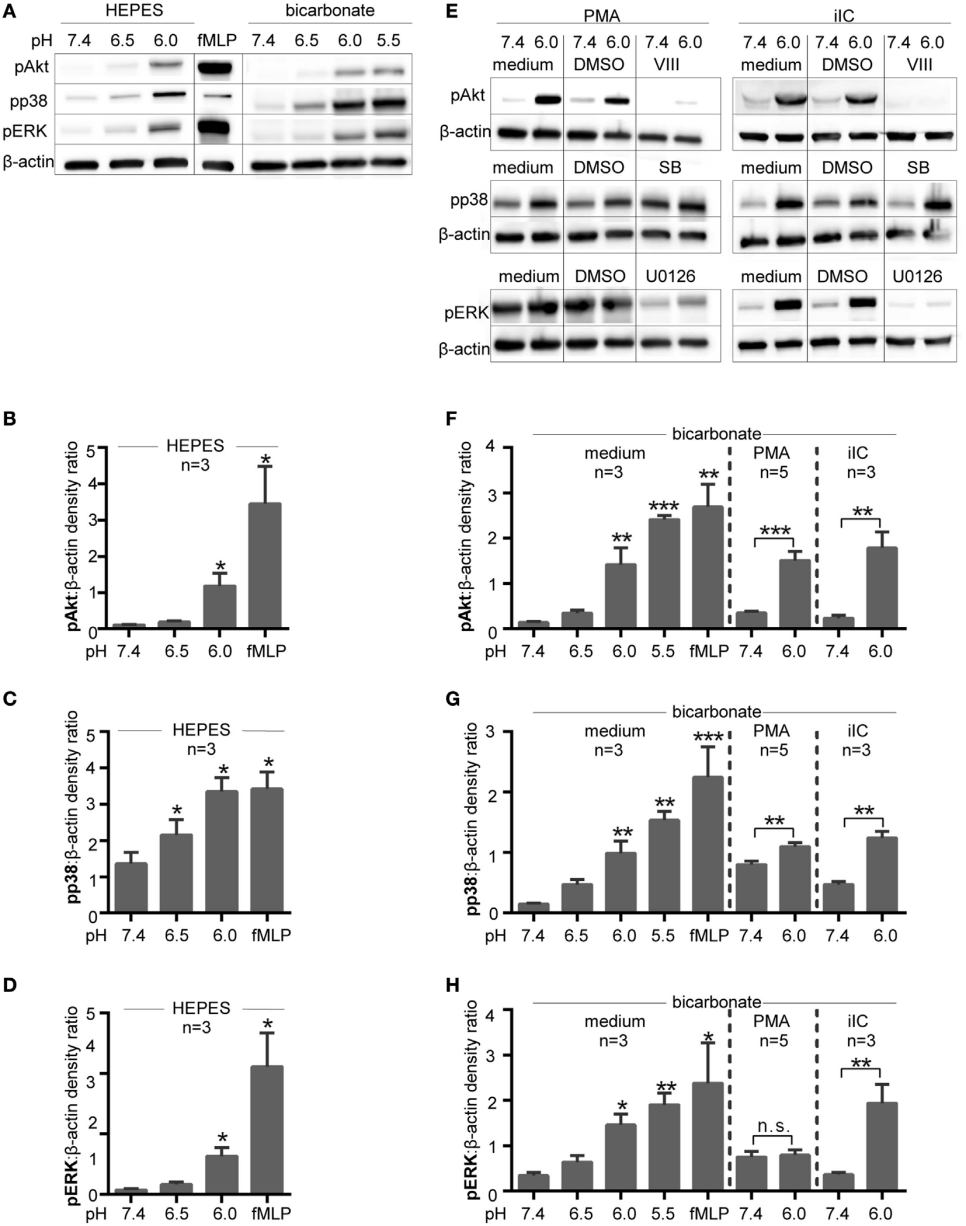
**Intracellular signaling pathways are activated under extracellular acidosis**. Phosphorylation of Akt, p38 MAPK, and ERK was analyzed by SDS-PAGE and western blots of whole cell lysates of neutrophils (5 × 10^6^ cells/ml). For quantification, the signals of pAkt, pp38 MAPK, and pERK were normalized to the β-actin signal detected on the same blot (*n* = 3–5). **(A)** Neutrophils were incubated for 15 min at various pH values in *N*-2-hydroxyethylpiperazine-*N*′-2-ethanesulfonic-acid (HEPES)- or bicarbonate-buffered media. Blots shown are representative of three independent experiments. **(B–D)** Show quantification for pAkt, pp38 MAPK, and pERK under HEPES-buffered conditions and **(F–H)** under bicarbonate-buffered conditions. *p < 0.05, **p < 0.01, and ***p < 0.001 as compared to unstimulated pH 7.4. **(E)** Neutrophils were preincubated for 15 min in bicarbonate-buffered media with 10 µM VIII (Akt inhibitor), 10 µM U0126 (MEK/ERK inhibitor), 10 µM SB203580 (p38 inhibitor), or solvent (dimethyl sulfoxide; 1:1,000) before stimulation for 15 min with phorbol myristate acetate (PMA) or immobilized immune complex (iIC). Blots shown in panel **(E)** are representative of two independent experiments.

As Akt, ERK, and p38 MAPK pathways are involved in the induction of PMA- and iIC-induced ROS-dependent NET release, phosphorylation of these molecules was assessed by western blot analysis at various pH values. This analysis revealed that extracellular acidosis alone leads to the phosphorylation of Akt, ERK1/2, and p38-MAPK in human neutrophils under both bicarbonate- and HEPES-buffered conditions (Figures 9A–D,F–H).

In order to avoid experimental artifact and to try to mimic *in vivo* conditions the CO_2_–bicarbonate-buffered system was used in subsequent signaling experiments. As expected, stimulation of neutrophils with PMA-induced phosphorylation of p38 MAPK and ERK and Akt (Figures 9E–H). Extracellular acidosis (pH 6.0) further enhanced phosphorylation of p38 MAPK and Akt in PMA-stimulated neutrophils (Figures 9E–G), while the PMA-induced ERK phosphorylation was apparently not further enhanced by acidosis (Figures 9E,H). iIC-induced phosphorylation of Akt, p38 MAPK, and ERK was also further enhanced under extracellular acidosis (Figures 9E,H).

To investigate whether the activation of Akt, ERK, or p38 MAPK pathways are involved in acidosis-dependent inhibition of NETosis, neutrophils were treated with U0126, VIII, or SB203580, inhibitors of MEK 1/2 upstream of ERK, Akt, and p38-MAPK, respectively. As expected, the phosphorylation of ERK1/2 was prevented by use of the inhibitor U0126 and the phosphorylation of Akt by the use of the Akt inhibitor VIII under both physiological (pH 7.4) and acidic (pH 6.0) conditions (Figure 9E). These data reveal that the inhibitors are functional under acidic conditions. As SB203580 inhibits the catalytic p38 activity by binding to the ATP pocket, this inhibitor does not inhibit the p38 phosphorylation by upstream kinases and no effect on phosphorylation was visible in western blot analysis (Figure 9E).

**Figure 10:**
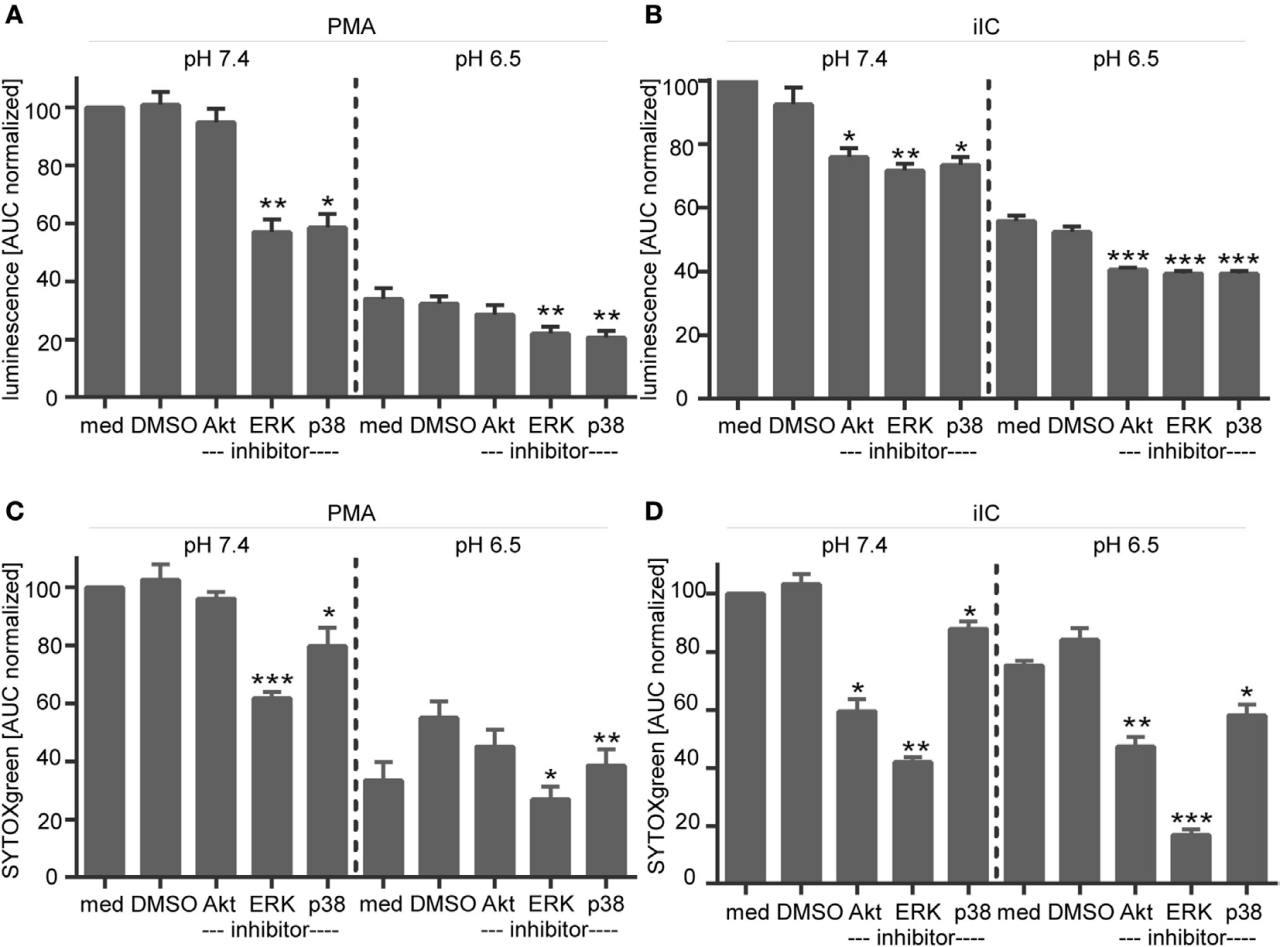
**Influence of signaling inhibitors on phorbol myristate acetate (PMA)- and immobilized immune complex (iIC)-induced reactive oxygen species (ROS) and neutrophil extracellular trap (NET) release under physiological and acidic conditions**. **(A,C)** Show ROS and **(B,D)** NET release for PMA- and iIC-stimulated neutrophils treated with signaling inhibitors. ROS production **(A,C)** was analyzed by the luminol assay over a periode of 1 h and NET release **(B,D)** was monitored by SYTOXgreen assay over a period of 4 h for PMA and 7 h for iIC stimulation. Normalized mean area under the curve (normalized AUC) values (mean ± SEM) of the luminol and SYTOX-assay are shown. AUC values were normalized to PMA or iIC-treated pH 7.4 samples without solvent or inhibitors (*n* = 3; *p < 0.05, **p < 0.01, ***p < 0.001 as compared to solvent control of the same pH value).

The inhibitor studies were performed at pH 7.4, 6.5, 6.0, and 5.5, but as no effects were observed at the different pH values, only results for pH 7.4 and 6.5 are shown. Upon PMA stimulation, we observed a diminished ROS production (Figure 10A) and NET release (Figure 10C), when ERK and p38 MAPK activity were inhibited by using U0126 and SB203580 under physiological pH. Although Akt is suggested to be involved in PMA-induced NETosis and we observed a slight phosphorylation of Akt upon PMA stimulation (Figures 9B,F), blocking of Akt phosphorylation did not affect the PMA-induced ROS and NET formation (Figures 10A,C). The same effects were observed under acidic conditions. For iIC-stimulated neutrophils, we observed, as expected, less ROS production and NET release under physiological conditions when Akt, ERK, or p38 MAPK were inhibited (Figures 10B,D). These inhibitory effects were also obvious under acidic conditions.

**Figure 11:**
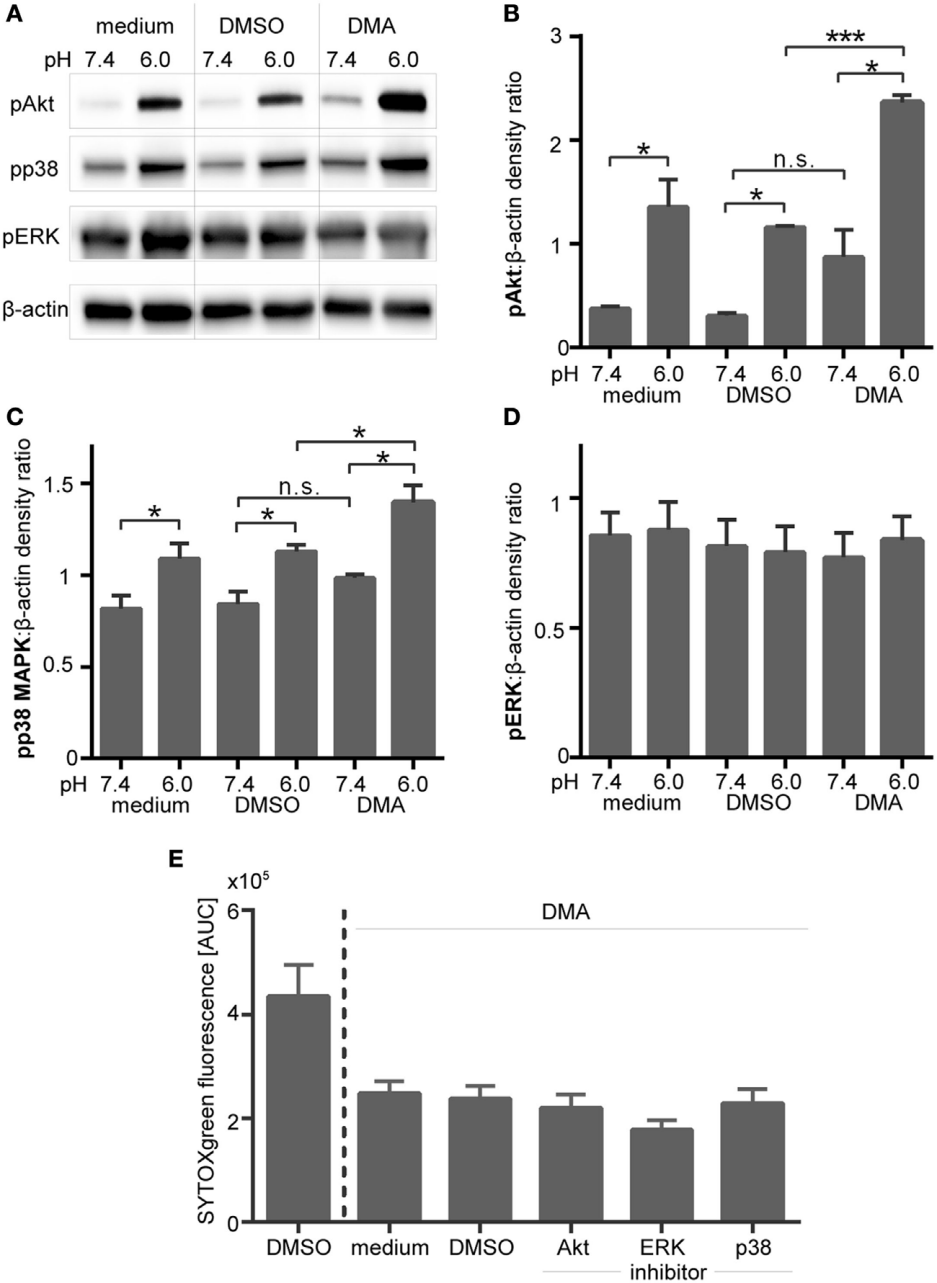
**Inhibition of NHE-1 results in enhanced phosphorylation of Akt and p38 MAPK in phorbol myristate acetate (PMA)-activated neutrophils**. Neutrophils were preincubated for 30 min in bicarbonate-buffered RPMI at pH 7.4 and 6.0 with the NHE-1 inhibitor (DMA, 10 µM), solvent control (dimethyl sulfoxide, 1:1,000), or left untreated (medium control) and were then stimulated for 15 min with PMA. **(A)** Phosphorylation of Akt, ERK 1/2, and p38 MAPK was analyzed in western blots. Equal loading was shown by reprobing with anti-human β-actin antibodies. Blots shown are representative of three independent experiments. **(B–D)** For quantification, the western blot signals of **(B)** pAkt, **(C)** pp38 MAPK, and **(D)** pERK were normalized to the β-actin signals detected on the same blot. *n* = 3; *p < 0.05, ***p < 0.001. **(E)** Neutrophils (10^6^ cells/ml in bicarbonate-buffered RPMI) were pretreated for 30 min with 10 µM DMA and then preincubated for further 30 min with inhibitors of Akt (10 µM VIII), MEK/ERK (10 µM U0126), p38 MAPK (10 µM SB203580), or solvent (DMSO, 1:1,000). Following stimulation with PMA, neutrophil extracellular trap release was monitored by SYTOXgreen assay over a period of 4 h. Area under the curve (AUC) values (mean ± SEM) of the SYTOXgreen fluorescence are shown (*n* = 3).

Intracellular acidification, induced by blocking of NHE-1 in PMA-stimulated neutrophils also led to enhanced Akt and p38 MAPK phosphorylation (Figures 11A–C), but did not affect ERK phosphorylation (Figures 11A,D). The activating effect on Akt and p38 phosphorylation was statistical significant under extracellular acidosis (pH 6.0) but not under physiological pH (Figures 11B,D). Blocking of Akt, ERK, or p38 MAPK did not overturn the DMA/pHi-dependent inhibition of NETosis, as measured by the SYTOXgreen assay (Figure 11E). As blocking of Akt, ERK, or p38 MAPK did not reverse the inhibitory effect of extra- or intracellular acidosis on ROS and NET formation, these results suggest that these pathways play no direct role for acidosis-dependent inhibition of PMA/iIC-induced ROS and NETs.

### Extracellular Acidosis Results in Reduced Glycolysis

**Figure 12:**
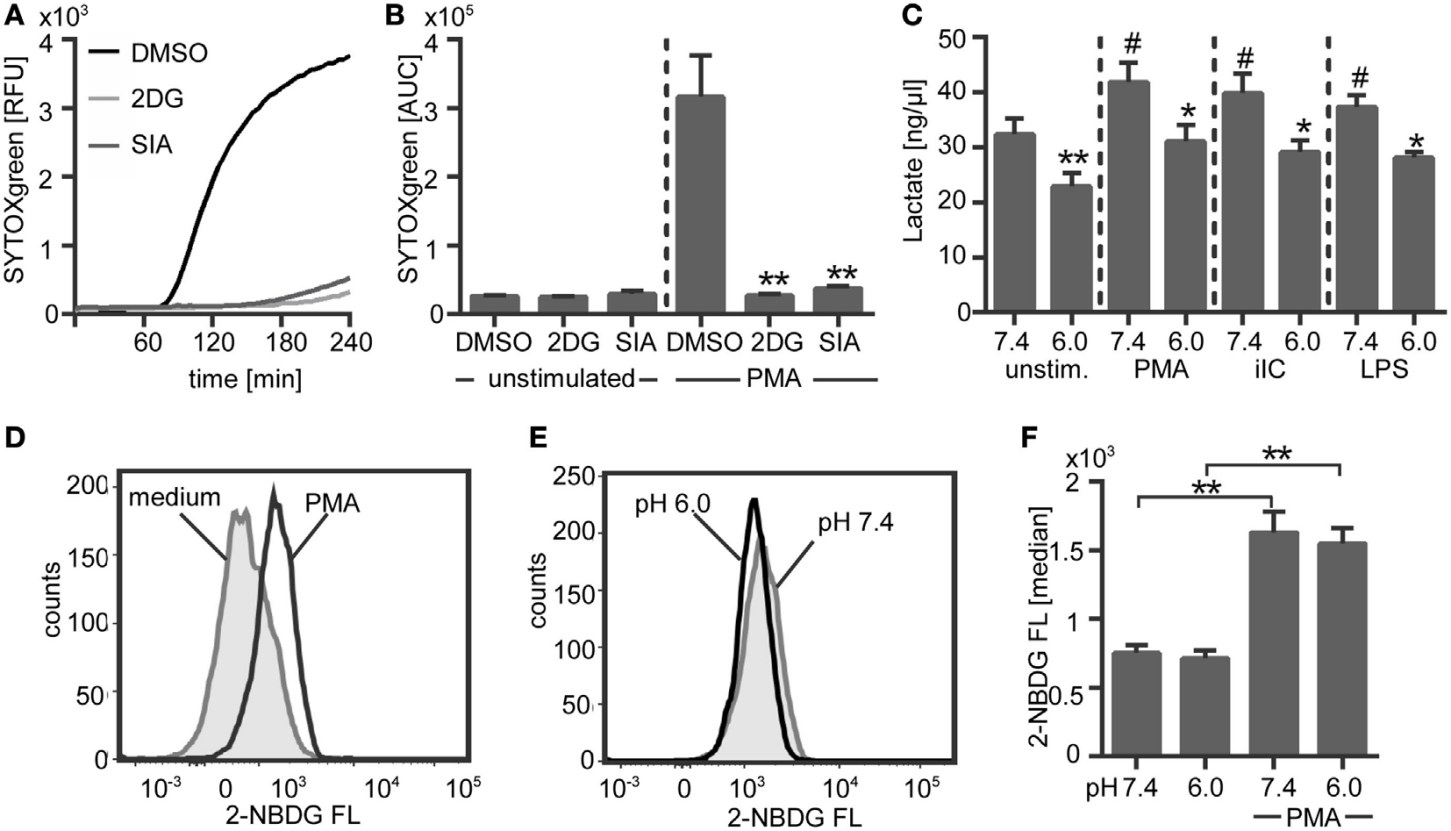
**NETosis depends on glycolysis and extracellular acidosis results in decreased glycolysis**. **(A,B)** 10^6^ neutrophils/ml were preincubated for 30 min under bicarbonate-buffered conditions at pH 7.4 with inhibitors of the glycolysis pathway [10 µM 2-deoxyglucose, 100 nM sodium idoacetate (SIA)] or solvent control (dimethyl sulfoxide, 1:1,000) and were then stimulated with phorbol myristate acetate (PMA). Release of neutrophil extracellular traps (NETs) was monitored for 4 h at 37°C by using the SYTOXgreen assays. **(A)** Representative real-time kinetics of NET release and **(B)** area under the curve (AUC) values (mean ± SEM) of NET-dependent relative fluorescence intensities (RFUs) as measured by the SYTOXgreen assay. *n* = 3, **p < 0.01 as compared to DMSO controls. **(C)** 5 × 10^6^ neutrophils/ml were preincubated for 30 min in bicarbonate-buffered medium at pH 7.4 and pH 6.0 and were then stimulated with PMA, immobilized immune complex (iIC), LPS, or left untreated. Lactate was detected in the culture supernatants by using a lactate assay kit and concentration of lactate was calculated after subtraction of the medium blanks (pH 7.4/6.0) by interpolation from standard curve. *n* = 3, *p < 0.05, **p < 0.01 as compared to pH 7.4, ^#^*p* < 0.05 as compared to unstimulated pH 7.4 sample. **(D,E)** 5 × 10^6^ neutrophils per milliliter were preincubated for 30 min in bicarbonate-buffered glucose-free medium at pH 7.4 and pH 6.0 and were then stimulated for 30 min with PMA or left untreated. The fluorescent glucose analog 2-NBDG was added for the last 10 min of incubation time and 2-NBDG uptake was analyzed by flow cytometry. Representative histograms showing the 2-NBDG fluorescent intensities of **(D)** unstimulated and PMA-stimulated neutrophils under physiological pH and **(E)** PMA-stimulated neutrophils under physiological versus acidic pH. **(F)** Median values ± SEM of the 2-NBDG fluorescence are given (*n* = 3, *p < 0.05, **p < 0.01).

To assess if NETosis depends on glycolysis, human neutrophils in bicarbonate-buffered medium were pretreated with two different inhibitors of the glycolysis pathway and the release of NETs was analyzed. Both inhibitors, 2DG, which inhibits the hexokinase and SIA, which inhibits the glyceraldehyde-3-phosphate dehydrogenase completely inhibited the NETosis (Figures 12A,B). These results clearly indicate that NETosis strictly depends on glycolysis. Next, we asked if glycolysis in neutrophils is altered in an acidic environment. The production of lactate, which is the end product of glycolysis, was measured in unstimulated and activated neutrophils under acidic (pH 6.0) versus physiological (pH 7.4) conditions in a CO_2_–bicarbonate-buffered system. Stimulation of neutrophils with PMA, iIC, or LPS resulted in a significant increase of glycolysis/lactate production as compared to unstimulated cells (Figure 12C). Extracellular acidosis led to a significant decrease in the glycolysis rate of both unstimulated and activated neutrophils (Figure 12C). As the production of NETs depends on glycolysis, we suggest that the inhibition of NETosis is the consequence of reduced glycolysis under acidic conditions.

Since glycolysis requires the presence of glucose, we performed a glucose-uptake assay to analyze if the decreased glycolysis rate under acidic conditions is due to a limited uptake of glucose. This assay was carried out by using the fluorescent glucose analog 2-NBDG. Stimulation of neutrophils with PMA induced a significant increase in 2-NBDG uptake (Figures 12D,F). The basal glucose uptake of unstimulated neutrophils was not affected by extracellular acidosis (Figure 12F). In PMA-activated neutrophils, we could observe a slight, but not statistically significant, decrease in glucose uptake under extracellular acidosis in comparison to physiological pH of 7.4 (Figures 12E,F). Thus, it is to expect that a limited glucose uptake is not the reason for reduced glycolysis and for the inhibition of NETosis under extracellular acidosis.

## Discussion

The inflammatory microenvironment under physiological and pathological conditions is commonly characterized by extracellular acidosis (pH <7.35) (21–23, 62). It has been reported that the functions of neutrophils are modulated by the extracellular pH (40). Although several functions of neutrophils have been analyzed under acidic conditions, to our knowledge, the effect of extracellular acidosis on the formation of NETs remains mainly unexplored. Only few studies suggest that the pH can modulate NETosis. A recent study describes that the CO_2_ to bicarbonate ratio modulates the spontaneous and induced NETosis, indicating that an acidic environment impairs NET formation (46). In this study, it was also observed that an alkaline pH (pH 7.6 and 7.8) can induce NET release under HEPES-buffered conditions (46). On the contrary, in another study acidic conditions of pH 7.0 and 6.5, enhanced PMA-induced NET release (45). Therefore, we designed a comprehensive study to elucidate the effect of extracellular acidosis on ROS-dependent PMA- and iIC-induced NETosis and to identify the underlying mechanisms. The study was performed in parallel in a CO_2_–bicabonate-buffered system, which mimics *in vivo* conditions and under HEPES-buffered conditions to verify the effects of pH independent of CO_2_ or bicarbonate. We could clearly show that extracellular acidosis (pH 6.5, 6.0, and 5.5) inhibits the release of PMA- and iIC-induced NETs in comparison to physiological pH 7.4. As an approach to manipulate the pHi, neutrophils were treated with DMA, an inhibitor of the Na^+^/H^+^ exchanger NHE-a. NHE-1 is the key regulator by which neutrophils restore physiological pHi, e.g., when they are activated by phorbol diesters (63) and blocking of NHE-1 in PMA-activated neutrophils was reported to result in intracellular acidification (33). Induction of intracellular acidosis by inhibition of NHE-1 also significantly reduced the ROS and NET release of PMA- and iIC-stimulated neutrophils, suggesting that NET production is also modulated by the intracellular pH. Our data suggest that the inhibition of NETosis under acidic conditions is due to the limited ROS production and restricted glycolytic capacity.

Phorbol myristate acetate is the best characterized and a strong inducer of NETosis (50). Since PMA acts directly on PKC, by using PMA, we avoid the complexity of various signaling pathways utilized by receptor-mediated stimuli upstream of PKC. iIC play a central role in the pathogenesis of several autoimmune inflammatory diseases as they provoke priming and activation of neutrophils and stimulate the release of NETs (8, 51). Both PMA and iIC induce a ROS-dependent lytic/suicidal NETosis, a form of cell death that is distinct from apoptosis or necrosis. Beside ROS-dependent NETosis, ROS independent NET release and vital NET formation, which allows neutrophils to stay viable, has been reported for few microorganisms and certain stimuli (5, 64–67). Since the mechanisms of ROS-dependent suicidal NETosis are better understood, in this study, we focused on the effect of extracellular acidosis on this form of NETosis.

NETosis is a multifactorial process, but detailed mechanisms are not completely understood so far. Both PMA- and iIC-induced NETosis are known to be ROS dependent (3–5, 8). In the present study, we have shown that extracellular acidosis as well as intracellular acidosis (induced by NHE-1 blocking) results in decreased intra- and extracellular ROS. Reduced ROS under extra- and intracellular acidosis was also described by others (39, 68, 69). The inhibition of NETosis under acidic conditions seems to be a direct consequence of limited ROS, as hydrogen peroxide treatment, which directly induces NETs (70–74), recovers NET formation of stimulated neutrophils under acidosis. ROS may have multifactorial functions in NETosis, which still need to be elucidated. In PMA-stimulated neutrophils, NOX2-dependent ROS have been shown to activate intracellular signaling cascades (Raf/MEK/ERK, Akt, and p38 MAPK), which mediate NETosis (7, 75) while activation of phosphatidylinositol 3-kinase (PI3K)/Akt, p38 MAPK, and ERK results directly from receptor activation and is NOX2/ROS independent upon iIC stimulation (8). We observed that extracellular acidosis alone, without additional stimuli, leads to the phosphorylation of ERK 1/2, p38 MAPK, and Akt. Moreover, PMA- and iIC-induced phosphorylation of these signaling molecules was enhanced under extracellular acidosis while ROS and NET production were inhibited. In addition, we could show enhanced phosphorylation of p38 MAPK and Akt when the pHi was acidified by blocking NHE-1. The acidosis-induced phosphorylation of Akt, p38 MAPK, and ERK, however, does not seem to play a direct role for acidosis-dependent inhibition of NETosis. This view is supported by the finding that inhibition of ERK 1/2, p38 MAPK, and Akt did not reverse the inhibitory effect of acidosis on PMA- or iIC-induced ROS and NET formation. Martinez et al. also showed that extracellular acidosis (pH 6.5) in a bicarbonate-buffered system triggers phosphorylation of Akt and ERK (44). By use of inhibitors, they could show that activation of these signaling pathways under acidic pH is responsible for neutrophil shape change, calcium mobilization, and enhanced endocytosis. Hence, they concluded that extracellular acidosis induces neutrophil activation *via* PI3K/Akt and ERK pathways. Under bicarbonate-buffered conditions, we measured a prolonged survival, delayed apoptosis, stable phagocytosis, and higher bacterial killing capacity under extracellular acidosis, indicating an enhanced functionality of neutrophils. We have not addressed, however, the role of p38 MAPK, ERK, and Akt in apoptosis delay and for neutrophil phagocytosis and killing capacity. Akt activation is also known to result in apoptosis inhibition (76). Also in PMA-stimulated neutrophils, activation of Akt has been shown to suppress apoptosis while inhibition of Akt promotes caspase-dependent apoptosis (75). Thus, we cannot rule out that upregulation of p38 MAPK, Akt, and ERK is linked to acidosis-dependent apoptosis delay and activation of neutrophils. But acidosis-dependent activation of p38 MAPK, Akt, and ERK does not seem to be involved in the inhibition of PMA- and iIC-induced ROS and NET production under acidic conditions.

Some studies suggest that ROS are important signal mediators that regulate the morphological changes observed during NETosis. In PMA-induced NETosis, hydrogen peroxide triggers the activation and dissociation of NE from the azurosome to the cytoplasm (11). NE is a key player during NETosis (10). In the cytoplasm, activated NE binds and degrades F-actin, liberating the protease to enter the nucleus where it drives the nuclear decondensation required for NET release (10, 11). As hydrogen peroxide directly regulates NE and actin dynamics and thus plays a key role for the morphological process of NETosis, acidosis-induced inhibition of ROS, therefore, could directly result in less NETosis. This hypothesis is supported by the fact that hydrogen peroxide treatment of neutrophils in an acidic environment recovers NET formation to the same amount as under physiological conditions.

It was also suggested that ROS may inactivate caspases, thereby inhibiting apoptosis and favoring autophagy. Interplay between autophagy and NET formation has been described for PMA-induced NETosis (77, 78) and for NETs in acute gout inflammatory arthritis (79), sepsis (80), and ANCA-associated vasculitis (81). Both ROS and autophagy are required for NETosis, as inhibition of either autophagy- or NOX2-dependent ROS production prevents NET formation (77). Although one study described that ROS production and autophagy occur independently during NETosis (77), several studies showed that ROS mediate the autophagy process (82–84), and it is suggested that the level of intracellular ROS determines whether the autophagy reaction ends in NETosis. Following this theory, limited ROS under acidosis could affect the autophagy process and thus inhibit the NETosis.

Nevertheless more experiments are needed to figure out what are the molecular mechanisms downstream of ROS production and upstream of NET formation under physiological conditions and to reveal why limited ROS under acidic conditions inhibits NETosis.

Neutrophils obtain most of their energy from glycolysis and glucose is the principal energy source of neutrophils (85, 86). It was reported that PMA-induced NET formation depends on glucose and glycolysis (12). By use of two glycolysis pathway inhibitors, we could verify that NETosis depends on glycolysis. Upon stimulation with PMA, iIC, and LPS, we observed an increased glycolysis rate in neutrophils. Moreover, we could show that extracellular acidosis leads to a decrease in glycolysis of unstimulated and activated neutrophils. These results suggest that NETosis under extracellular acidosis is inhibited due to a limited glycolytic activity of neutrophils. As glycolysis depends on glucose, we performed a glucose-uptake assay to analyze whether a limited uptake of glucose may be the reason for a decreased glycolysis in an acidic environment. We could observe an enhanced uptake of glucose upon PMA stimulation. But, neither the basal glucose uptake of unstimulated cells nor the glucose consumption of PMA-stimulated cells was significantly altered under extracellular acidosis. These findings indicate that acidosis-dependent inhibition of glycolysis and NETosis is not due to a limited glucose uptake.

We observed a clear inhibitory effect of acidosis on PMA- and iIC-induced ROS-dependent NETosis by using two different buffer conditions, while the phagocytic activity and bacterial killing capacity of neutrophils was not inhibited. Regarding the modulatory effect of extracellular acidosis on neutrophil immune response, both inhibitory (39–43) and activating effects (38, 44) have been described. The biochemical basis of neutrophil activation or inhibition by extracellular acidosis is not yet clarified. Trevani et al. (38) postulate that the activating effects they observed on calcium mobilization, upregulation of CD18, MPO release, and enhanced H_2_O_2_ production are dependent on the presence of extracellular bicarbonate. By addition of HCl (to induce/adjust acidosis), bicarbonate reacts with protons (H^+^) to carbon dioxide (CO_2_) and water (H_2_O). As the lipid bilayer is not permeable for protons, but for CO_2_, diffusion of CO_2_ into the cell allows a rapid change of pHi (87). This drop in pHi is suggested to play a key role for neutrophil activation through extracellular acidosis. By using bicarbonate-free PBS, Trevani et al. observed a slight decrease in pHi after extracellular acidification by HCl (38) and they further suggest that, in HEPES-buffered conditions, addition of HCl may lead to titration of the HEPES without significantly affecting the pHi. Although this explanation sounds conclusive, there are other studies using bicarbonate-buffered conditions observing an inhibitory effect of extracellular acidosis on neutrophil functions such as oxidative burst and bacterial killing (39). We performed a comprehensive study under both HEPES-buffered conditions and in a CO_2_–bicarbonate-buffered system, which mimics *in vivo* conditions. Under both conditions, we observed a clear inhibitory effect of extracellular acidosis on PMA- and iIC-induced ROS and NET production. Also, intracellular acidification had an inhibitory effect under both bicarbonate- and HEPES-buffered conditions. These results suggest that the observed inhibitory effects on neutrophils ROS and NET production under acidic conditions are due to the acidic pH and independent of the chosen buffer system, as speculated by others (38). However, we have to consider that the biochemical composition of the media and CO_2_ level can affect neutrophil functions. Our present observations and data from others (46, 88) indicate that changes of each component of the bicarbonate system, namely, the pH, CO_2_ concentration, or bicarbonate concentration, can modulate NET release of neutrophils. We observed that unstimulated neutrophils can spontaneously release NETs under physiological pH 7.4 when they are incubated at room air in a bicarbonate-buffered medium. Increasing CO_2_ concentrations suppress the bicarbonate-induced NET signal and reduce PMA and iIC-induced NETosis. A recent study also described the potential of bicarbonate and CO_2_ to modulate NETosis by showing that a high ratio of bicarbonate to CO_2_ and a moderately alkaline pH enhance NETosis (46). Also, other functions of neutrophils such as ROS production or cytokine secretion are affected by CO_2_, pH, or bicarbonate (33, 87, 88). This sensitivity of neutrophils to pH and to the bicarbonate to CO_2_ ratio indicates how well neutrophils are adapted to versatile *in vivo* conditions. Blood pH is maintained by the bicarbonate system. Under physiological/healthy conditions, which are defined by pH 7.35–7.45, pCO_2_ 23–46 mmHg (5–6%), and HCO_3_ 21–26 mM, the spontaneously release of NETs is suppressed and thus the release of toxic neutrophil components/NETs, which can cause tissue damage, is prevented. The inflammatory microenvironment is characterized by extracellular acidosis (21–23, 62). Sensitivity to pH, CO_2_, or bicarbonate concentrations allows neutrophils to react to pathological changes and to detect inflamed areas. It is suggested that neutrophils can sense the border of inflamed areas by the pH gradient and that the drop in pH serves as an indicator for the progress of inflammation (46). Following this hypothesis, our data indicate that a highly inflammatory environment correlated with severe acidosis results in inhibition of extracellular operating effector mechanisms of neutrophils such as release of ROS and NET to avoid release of toxic components and tissue damage. Moreover, as the phagocytosis was stable and the bacterial killing capacity enhanced under extracellular acidosis, while NETosis is inhibited, we hypothesize that the main antimicrobial effector mechanism under acidic conditions is the intracellular killing of pathogens.

## Conclusion

Sensitivity to pH, CO_2_ and bicarbonate allows neutrophils to react to pathological chances and to detect inflamed areas. We here report functional sensitivity of ROS-dependent NETosis to extracellular and intracellular pH under HEPES- and bicarbonate-buffered conditions. Our findings suggest that the diminished NET release is a consequence of reduced glycolysis and reduced ROS production under acidic conditions. As ROS and NETs play a pathophysiological role in several human diseases, including autoimmunity and infectious diseases, sepsis, diabetes, acute lung injury, eclampsia, and thrombosis, clarification of mechanisms leading to acidosis in local microenvironments would be necessary to understand the pathophysiology of NET-dependent inflammatory diseases.

## Author Contributions

MB designed the study, performed research, analyzed the data, interpreted the results, performed statistical analysis, and wrote the manuscript. SM performed several studies and gave technical support. AB performed phagocytosis assays and SEM preparates. MK contributed scanning electron microscopy. TL participated on the interpretation of the results and contributed to writing the manuscript.

## Conflict of Interest Statement

The authors declare that the research was conducted in the absence of any commercial or financial relationships that could be construed as a potential conflict of interest.

## Abbreviations

AUC, area under the curve; DPI, diphenyleniodiniumchlorid; DMSO, dimethyl sulfoxide; SM, scanning electron microscopy; FM, fluorescence microscopy; GPI, glycosylphosphatidylinositol; HEPES, *N*-2-hydroxyethylpiperazine-*N*′-2-ethanesulfonic-acid; HSA, human serum albumin; IC, immune complexes; iIC, immobilized immune complexes; FL, fluorescence; MPO, myeloperoxidase; NETs, neutrophil extracellular traps; NOX2, NADPH oxidase; OD, optical density; PI, propidium iodide; PI3K, phosphatidylinositol 3-kinase; PMA, phorbol myristate acetate; PMN, polymorphonuclear neutrophil granulocytes; ROS, reactive oxygen species.
