# Supplementary material for: Extracellular Acidification Inhibits the ROS-Dependent Formation of Neutrophil Extracellular Traps
Source: Front Immunol. 2017 Feb 28;8:184. doi: 10.3389/fimmu.2017.00184 (PMC5329032; doi:10.3389/fimmu.2017.00184)
Supplement: Supplementary file 1 [file Image_1.PDF]

Supplemental 1

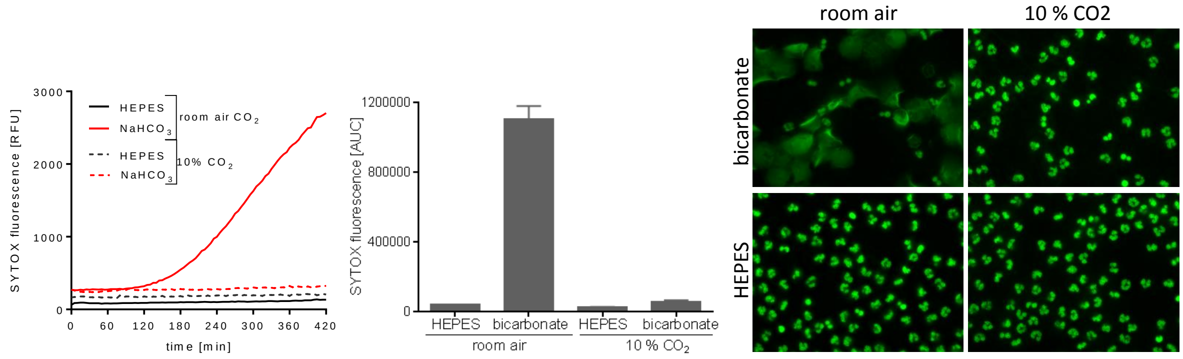

A B C

Supplemental Figure S1: $NaHCO_3$ induces NETs while CO_2 inhibits NET formation by human primary neutrophils. Neutrophils (10^6 cells/ml) were incubated for 7 h at 37°C in bicarbonate (26 mM $NaHCO_3$) or HEPES (20 mM) buffered RPMI1640 under room air CO_2 or 10 % CO_2. **(A)** representative real time kinetics and **(B)** AUC (mean ±SEM) of NET-dependent relative fluorescence intensities (RFU) as measured by the SYTOXgreen assay. **C)** representative fluorescence microscopy images of fixed and SYTOXgreen stained cells.
