# Supplementary material for: Extracellular Acidification Inhibits the ROS-Dependent Formation of Neutrophil Extracellular Traps
Source: Front Immunol. 2017 Feb 28;8:184. doi: 10.3389/fimmu.2017.00184 (PMC5329032; doi:10.3389/fimmu.2017.00184)
Supplement: Supplementary file 2 [file Image_2.PDF]

Supplemental 2

A

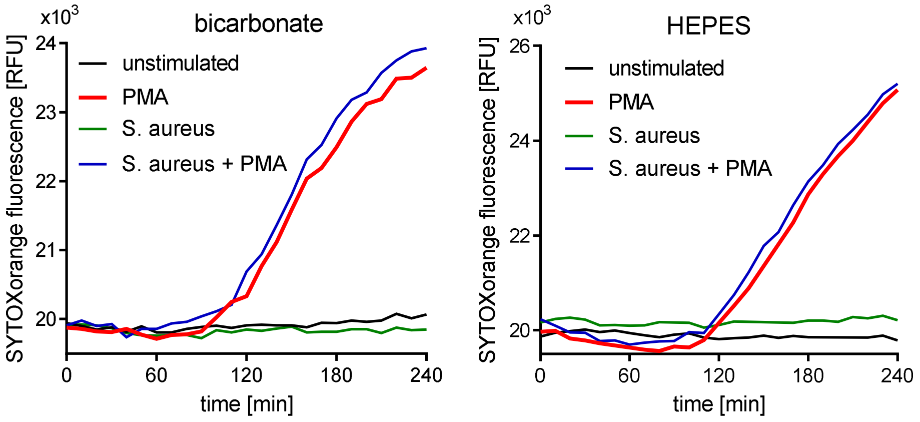

B

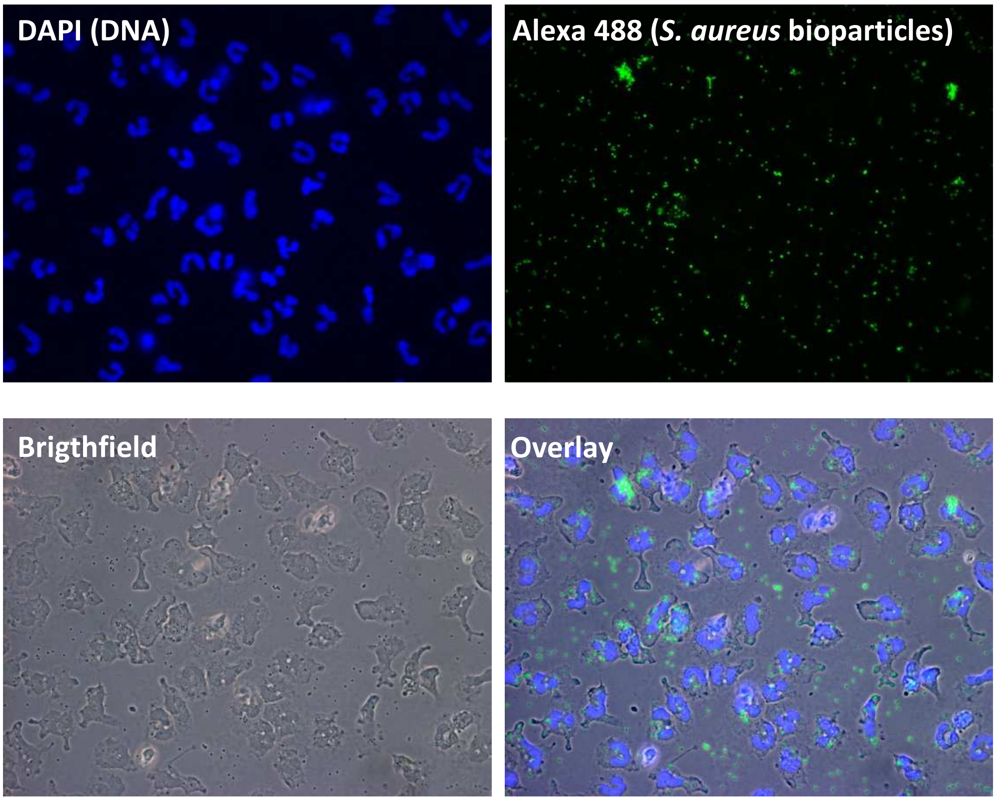

Supplemental 2: Opsonized *S. aureus* bioparticles do not induce the formation of NETs. Neutrophils were pre-incubated in bicarbonate or HEPES buffered medium at pH 7.4 following co-incubation with nonviable opsonized *S. aureus* bioparticles at 37°C under CO_2 atmosphere. **(A)** Real time kinetics of NET-dependent relative fluorescence intensities (RFU) of SYTOXorange are shown over a periode of 4 h incubation at 37°C. *S. aureus* bioparticles did not induce a SYTOX signal (NET release), but neutrophils treated with *S.aureus* were still able to produce NETs upon PMA stimulation. **(B)** Fluorescence microscopy images of fixed and DAPI (blue, DNA) stained co-cultures of neutrophils with Alexa-488 conjugated *S.aureus* bioparticles (green). The neutrophils exhibit a spreaded/activated morphology and phagocytosed the bioparticles but did not release DNA/NETs. The nucleus of all cells remains intact and shows typical polymorphonuclear morphology.
