# Supplementary material for: Extracellular Acidification Inhibits the ROS-Dependent Formation of Neutrophil Extracellular Traps
Source: Front Immunol. 2017 Feb 28;8:184. doi: 10.3389/fimmu.2017.00184 (PMC5329032; doi:10.3389/fimmu.2017.00184)
Supplement: Supplementary file 3 [file Image_3.PDF]

Supplemental 3

A B

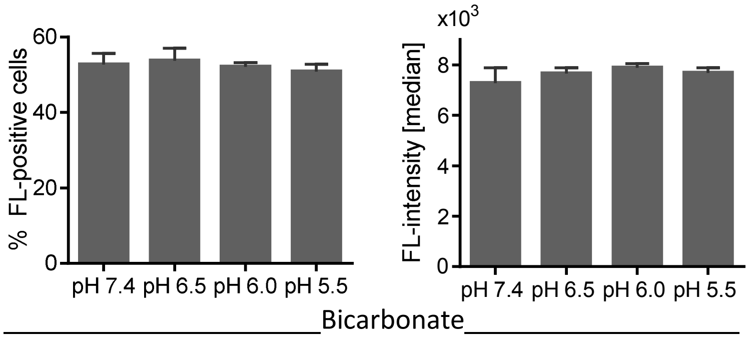

C D

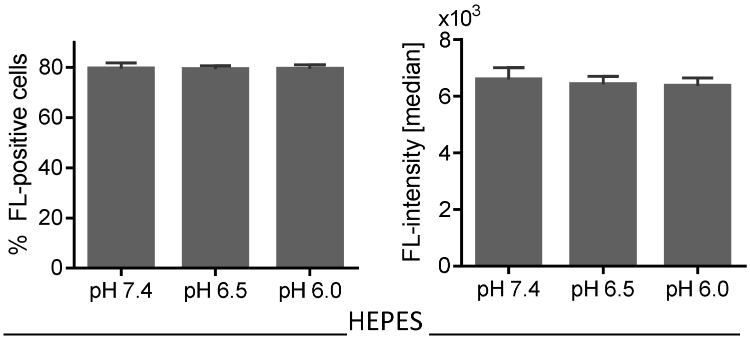

Supplemental 3: Extracellular acidosis does not affect the phagocytosis of FluoSphere beads. Neutrophils were pre-incubated in **(A, B)** bicarbonate or **(C, D)** HEPES buffered medium at pH 7.4, 6.5, 6.0 or 5.5 following co-incubation with FluoSpheres latex beads for 30 min at 37°C. Phagocytosis of beads was assessed by flow cytometry. **(A, C)** the percentage of neutrophils that phagocytosed beads (mean ± SEM) and **(B, D)** the mean fluorescence per cell (median ± SEM, right panel) are shown of three independent experiments.
