# Supplementary material for: Extracellular Acidification Inhibits the ROS-Dependent Formation of Neutrophil Extracellular Traps
Source: Front Immunol. 2017 Feb 28;8:184. doi: 10.3389/fimmu.2017.00184 (PMC5329032; doi:10.3389/fimmu.2017.00184)
Supplement: Supplementary file 4 [file Image_4.PDF]

Supplemental 4

A

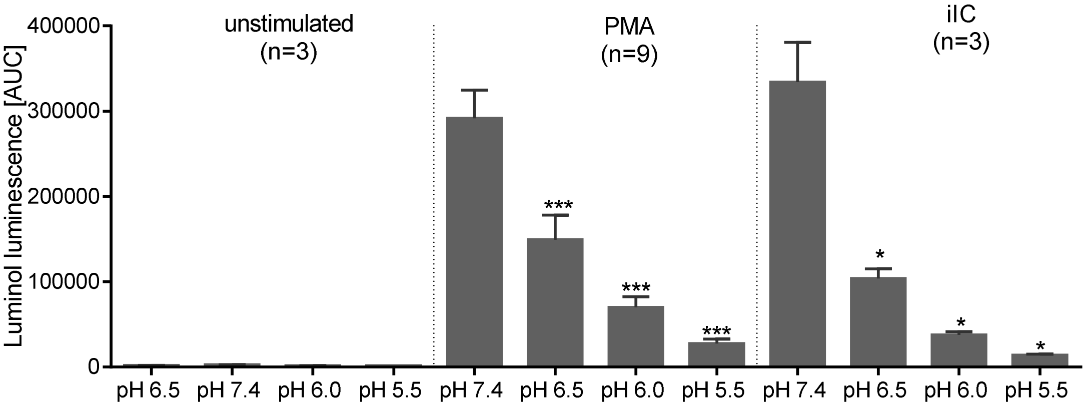

B

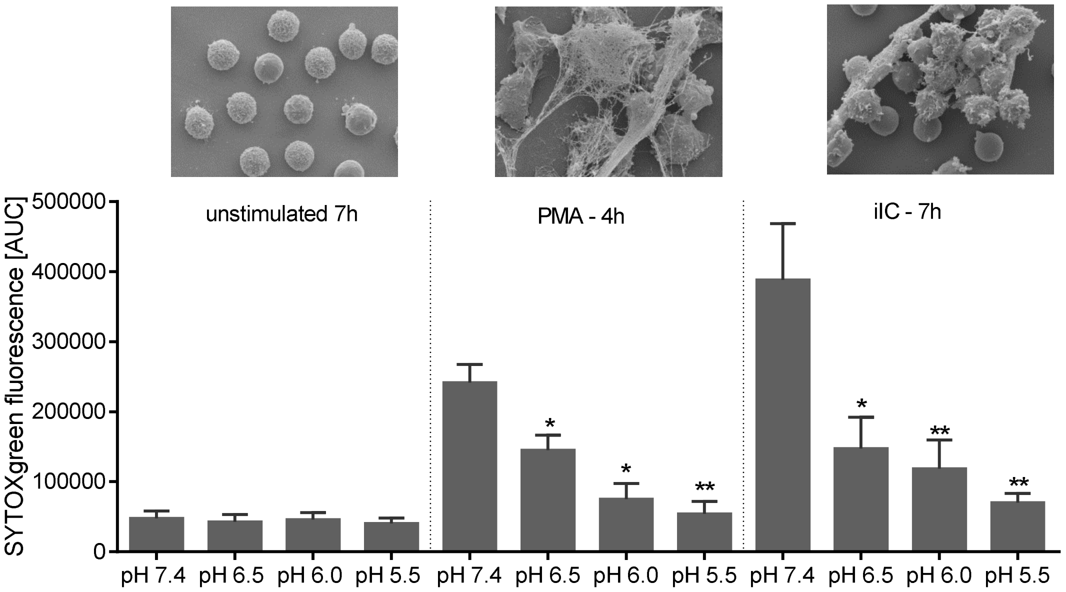

Supplemental 4: Extracellular acidosis inhibits the production of ROS and NETs under HEPES + $NaHCO_3^-$ buffered conditions. Neutrophils (2 x 10^6/ml for luminol assay and 10^6/ml for NET-assay) were preincubated for 30 min (37°C, 5 % CO_2) in RMPI1640 containing 10 mM HEPES and 2,2 g/l $NaHCO_3$ at pH 7.4, 6.5, 6.0 and 5.5. Cells were then stimulated with 20 nM PMA, iIC or left untreated and production of ROS and NETs was measured by using the luminol- and SYTOXgreen assays. Real time analysis for ROS was monitored for 1 h and for NETs for 4 h (PMA) or 7 h (iIC) at 37°C under 5 % CO_2. **(A)** show the area under the curve (AUC) values (mean ±SEM) of ROS-dependent chemiluminescence intensities and **(B)** NET-dependent relative fluorescence intensities as measured by the SYTOXgreen assay. n = 3-9, * $p < 0.05$, ** $p < 0.01$, *** $p < 0.001$ as compared to dedicated pH 7.4 samples. Scanning electron images shown in (B) are from unstimulated, PMA- and iIC stimulated neutrophils in HEPES+NaHCO3 buffered medium at pH 7.4.
