# Supplementary material for: Extracellular Acidification Inhibits the ROS-Dependent Formation of Neutrophil Extracellular Traps
Source: Front Immunol. 2017 Feb 28;8:184. doi: 10.3389/fimmu.2017.00184 (PMC5329032; doi:10.3389/fimmu.2017.00184)
Supplement: Supplementary file 5 [file Image_5.PDF]

Supplemental 5

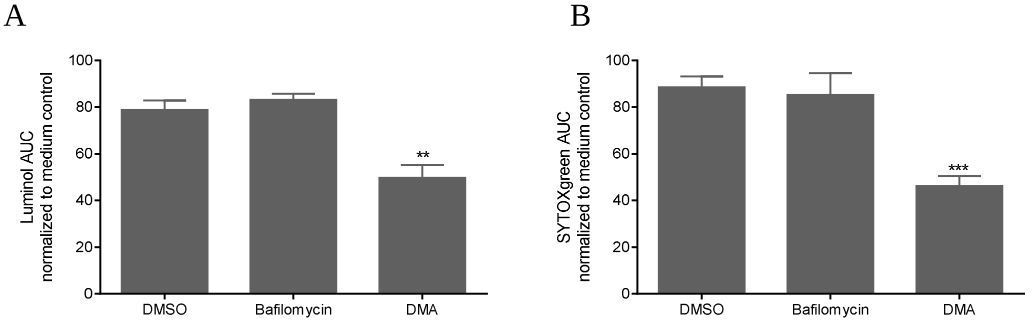

Supplemental 5: Inhibition of NHE-1 leads to a reduced production of PMA-induced ROS and NETs in HEPES+NaHCO$_3$ buffered medium. Neutrophils (2 x 10^6/ml for ROS assays, 10^6/ml for NET-assay) were pre-incubated for 30 min in HEPES+NaHCO$_3$ buffered RPMI1640 at pH 7.4 with inhibitors of NHE-1 (DMA, 10µM), V-ATPase (Bafilomycin, 100 nM), solvent control (DMSO, 1:1000) or left untreated (medium control) and were then stimulated with PMA. Release of ROS was monitored for 1 h and release of NETs for 4 h at 37°C (under 5 % CO_2) by using the luminol- and the SYTOXgreen assays. **(A)** normalized area under the curve (AUC) values (mean ± SEM) of ROS-dependent luminol-chemiluminescence and **(B)** of NET-dependent fluorescence intensities as measured by the SYTOXgreen assay. AUC values were normalized to PMA-stimulated medium control (PMA stimulated neutrophils in medium pH 7.4 without solvent or inhibitors) n = 3, **p<0.01, ***p<0.001 as compared to DMSO control.
