# Supplementary material for: Extracellular Acidification Inhibits the ROS-Dependent Formation of Neutrophil Extracellular Traps
Source: Front Immunol. 2017 Feb 28;8:184. doi: 10.3389/fimmu.2017.00184 (PMC5329032; doi:10.3389/fimmu.2017.00184)

Original full western blot images for Fig. 9 A

unstimulated in HEPES and bicarbonate

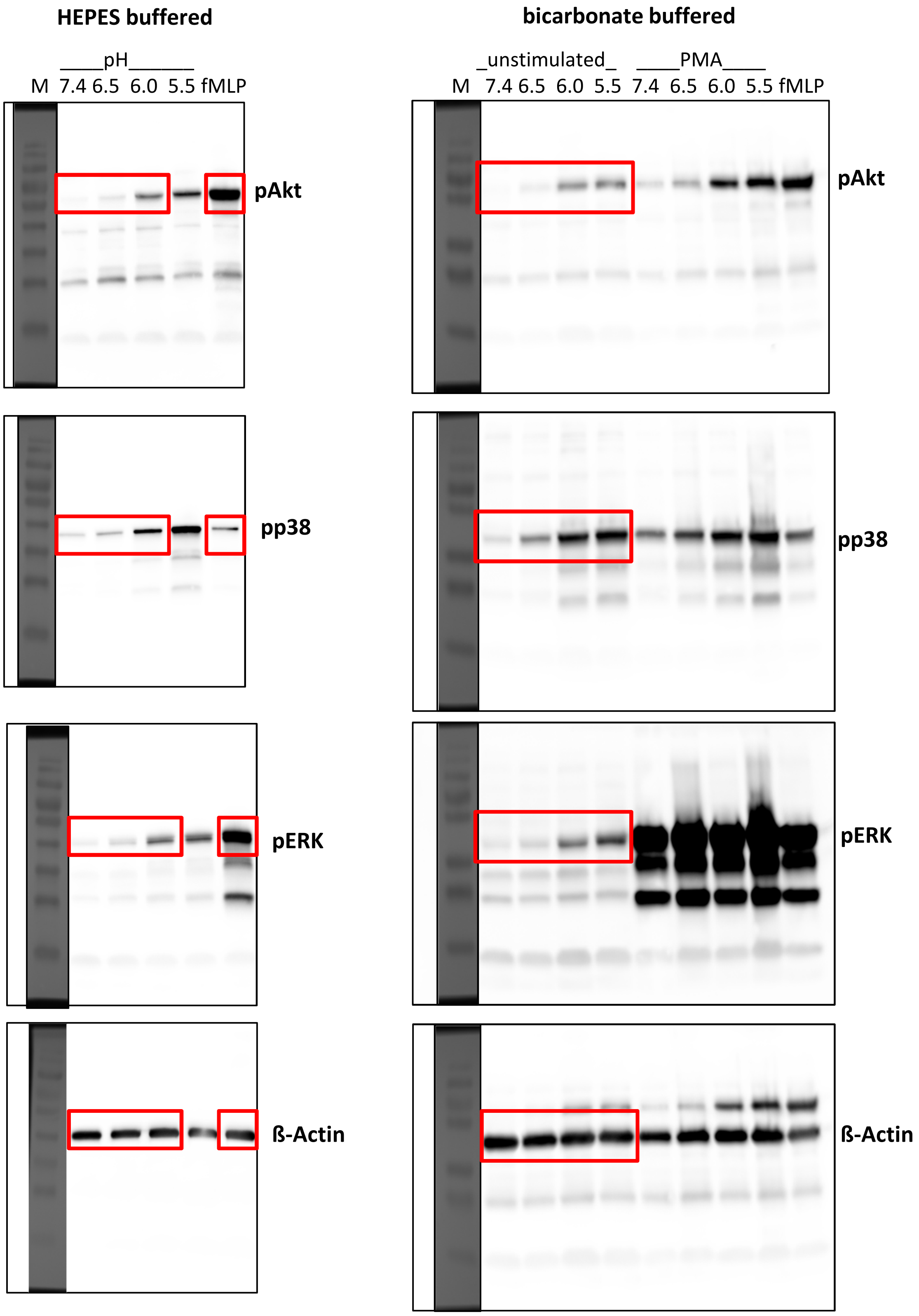

Original full western blot images for Fig. 9 B

PMA stimulation, bicarbonate buffered

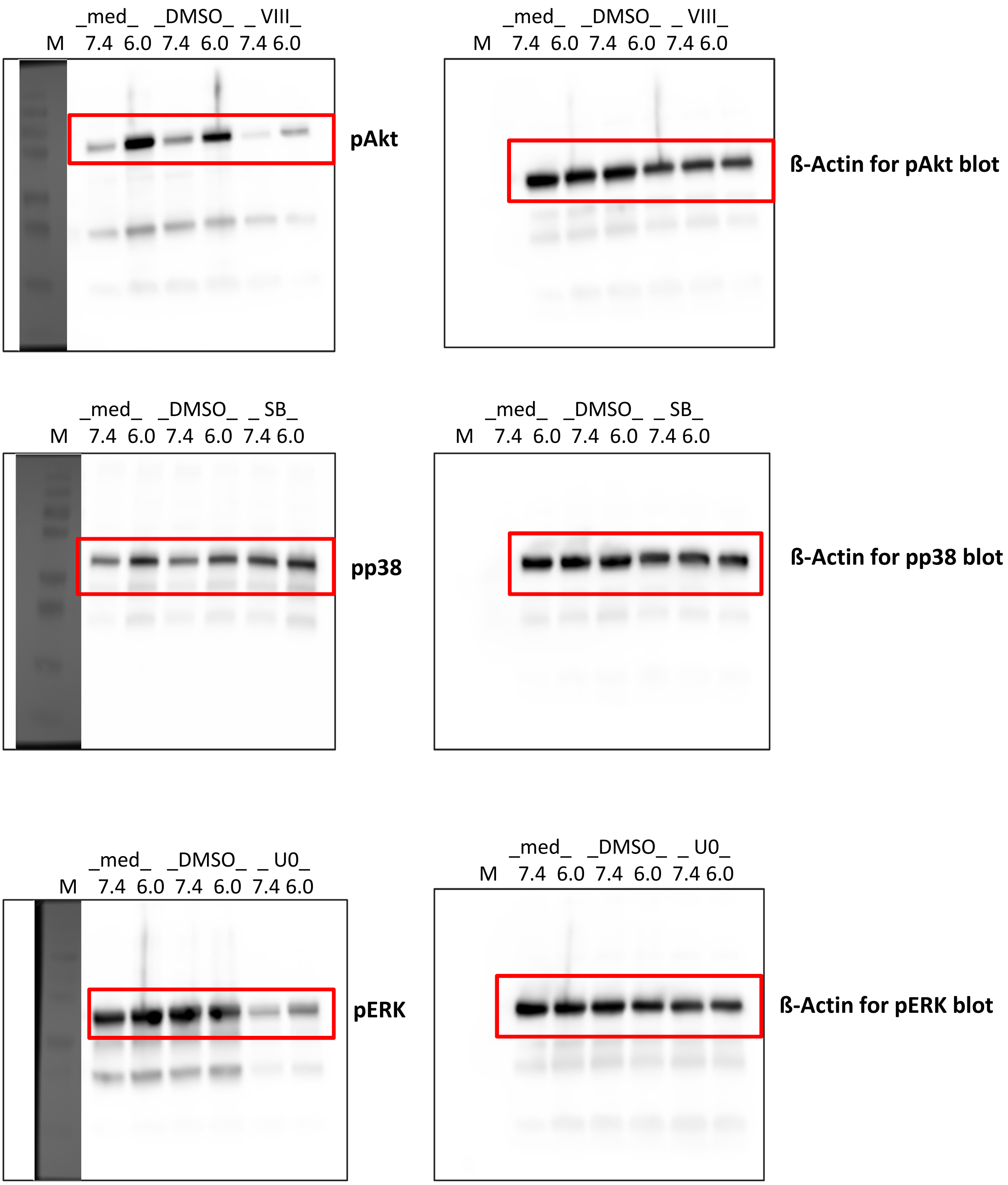

Original full western blot images for Fig. 9 B

iIC stimulation, bicarbonate buffered

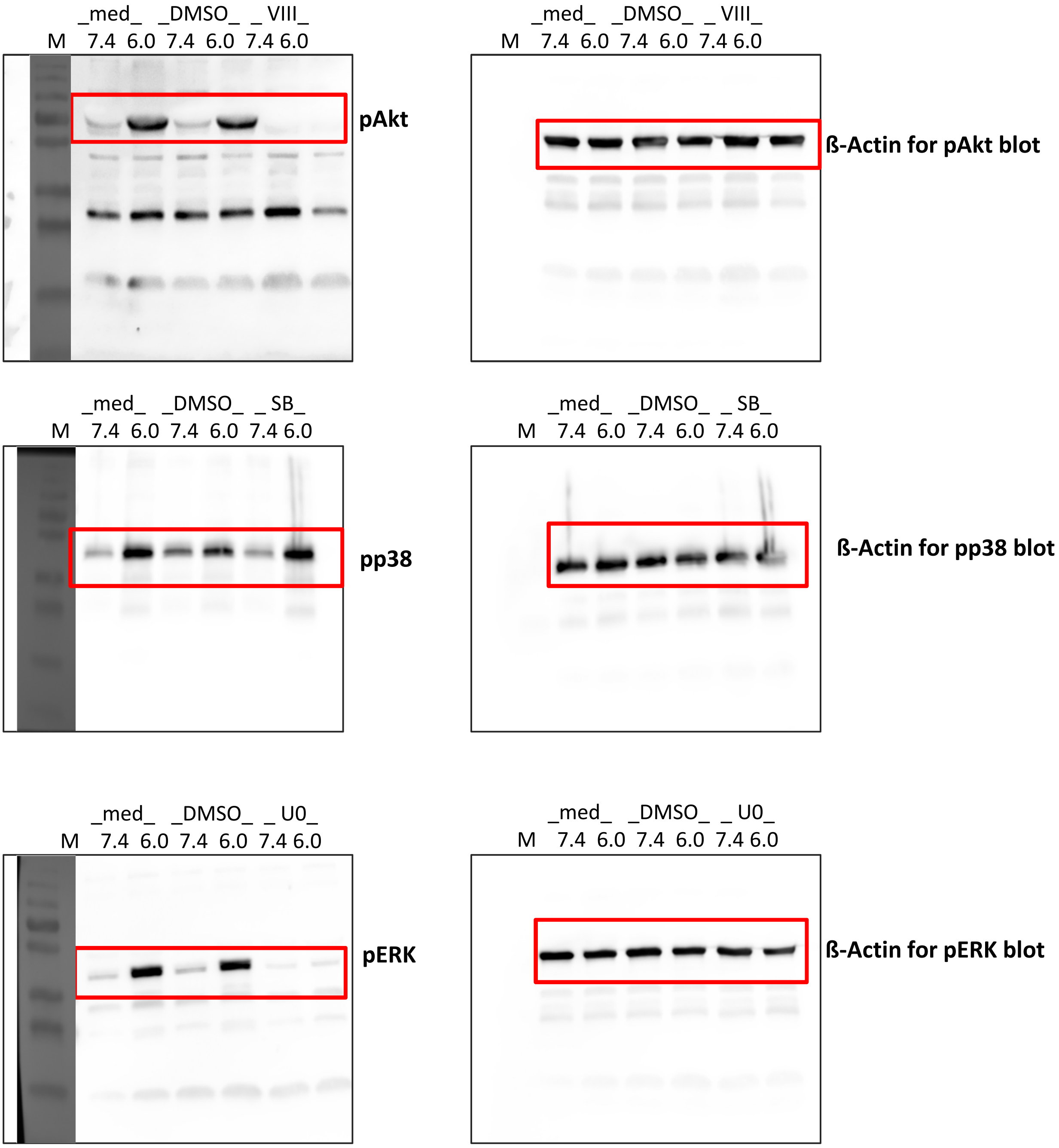

Original full western blot images for Fig. 11 A

PMA stimulated, bicarbonate buffered

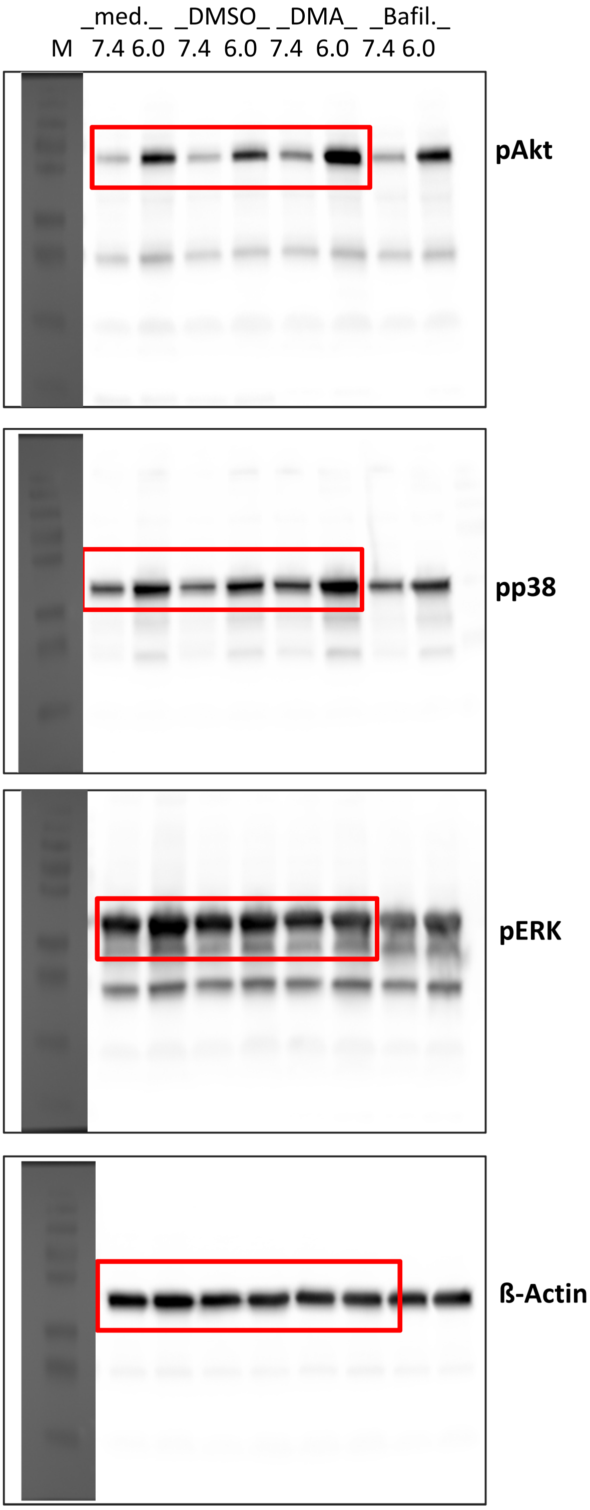

Supplement: Supplementary file 6 [file Image_6.PDF]
